# Research on fault prediction and speed control system for unmanned combine harvesters based on IPSO-SVM and fuzzy logic

**DOI:** 10.3389/fpls.2025.1577175

**Published:** 2025-06-03

**Authors:** Shaocen Zhang, Chongquan Zang, Zhang Yang, Lingyu Tang, Kun Wang, Anzhe Wang, Wenming Chen, Qi Song, Xinhua Wei

**Affiliations:** ^1^ School of Agricultural Engineering, Jiangsu University, Zhenjiang, China; ^2^ Key Laboratory for Theory and Technology of Intelligent Agricultural Machinery and Equipment, Jiangsu University, Zhenjiang, China

**Keywords:** unmanned harvester, fault prediction, IPSO-SVM, fuzzy control, clogging prevention, slip rate monitoring, intelligent farming, machine learning

## Abstract

This study proposes an IPSO-SVM-based fault prediction and fuzzy speed control system for unmanned combine harvesters. The primary goal is to prevent clogging failures and ensure long-term stable operation of unmanned harvesting machines, maintaining efficiency while minimizing downtime. The system integrates multi-component slip rate data, collected from critical parts of the harvester, and uses the IPSO-SVM model for fault warning. The fuzzy control algorithm adjusts the operating speed based on the predicted fault status and feeding rate to mitigate clogging risks. Experimental results show that the system can accurately identify over 98.5% of fault states and reduce the occurrence of complete blockage by adjusting the harvester’s speed within 0.5 to 2 seconds after minor clogging. This work demonstrates the feasibility of applying the system in field environments, providing a reliable solution for the intelligent and unmanned operation of combine harvesters in fields.

## 1 Introduction

The rice-wheat rotation has a tight planting schedule and limited labor availability, leading to widespread poor tillage and planting, as well as poor working conditions under harsh weather conditions (An et al., 2018; Marinoudi et al., 2019). Among the various steps in the “tillage, sowing, management, and harvesting” process, the quality of harvesting operations is particularly critical, involving relatively complex tasks and rigid labor requirements Lowenberg-DeBoer et al., 2020). The development of intelligent and unmanned combine harvesters not only addresses labor shortages but also enhances land utilization and improves agricultural machinery efficiency (Boursianis et al., 2022; Elijah et al., 2018). Currently, commercial agricultural machinery auxiliary driving systems are relatively mature, assisting operators in controlling the steering wheel and ensuring path tracking for field operations (Li et al., 2023, 2022; Lu et al., 2020). However, the operator still needs to handle other fieldwork mechanisms. For example, during harvesting operations, the operator must adjust the vehicle’s speed to avoid clogging based on the load, modify the header height according to terrain and crop lodging, and adjust the reel height according to the crop’s ear position (Yanxin et al., 2022). Currently, these auxiliary systems save labor but do not reduce labor costs effectively (Tey and Brindal, 2022), making the development of an operational control system a key step toward achieving unmanned agricultural machinery (Chen et al., 2023; Jin et al., 2021). This study focuses on the operational load speed control system of rice-wheat combined harvesters.

Scholars at home and abroad have combined information and intelligent technologies to study the state monitoring and fault diagnosis of combine harvesters, achieving significant results (Qiu et al., 2022; Liang et al., 2016; Xu et al., 2019). Wang et al. (2023) pointed out that the threshing system, being one of the key components of a harvester, is easily influenced by crop characteristics, feeding rates, and the harvester’s forward speed. When the feeding rate exceeds the threshing drum’s power match, the drum speed decreases, and the drive belt may slip, potentially causing a clogging failure that damages related components. Craessaerts et al. (2010a) and Yang et al. (2024) categorized common harvester faults into header faults, threshing system faults, and re-threshing faults, analyzing the causes of these faults and proposing preventive measures to reduce their occurrence. Pavlyuk et al. (2022) analyzed the main system performance parameters of combine harvesters, identifying failure-prone areas such as the harvesting section, mechanical drive section, and threshing section, and determined the fault distribution during harvesting operations through experiments.

Several studies have attempted to control the harvester’s load via feedback from key component working conditions, stabilizing the harvesting state to avoid failure (Chen et al., 2017; Li et al., 2022; Ma et al., 2023; Yu et al., 2024). Kruse et al. (1983) used changes in engine load to represent the harvester’s load, monitoring the engine’s torque as feedback to adjust the travel speed of the full-feeding combine harvester, keeping the feeding rate stable. McGechan and Glasbey (1982) used threshing drum torque, main auger torque, and loss rate as reference variables to adjust speed, maintaining a stable operating state for the harvester. Li et al. (2021) developed an online monitoring system for the harvester’s hydraulic components, such as the header, conveyor, and threshing drum, based on LabVIEW, enabling real-time collection of key working parameters and fault warnings. Abdeen et al. (2022) designed a stress monitoring system for the threshing drum’s top cover using resistive sensors, achieving real-time monitoring of feeding rates and early warnings of threshing drum clogging. Qin et al. (2011) designed a harvesting speed control system based on RBF, using header auger speed to assist in feeding state measurement and maintaining a constant threshing drum speed.

With the widespread application of machine learning algorithms in fault diagnosis, more solutions are available for diagnosing faults (Hao et al., 2022; Chen et al., 2020). Craessaerts et al. (2010b) suggested that using historical data from combine harvesters and artificial neural networks to construct fault diagnosis models can achieve approximately 80% fault recognition accuracy. By integrating fuzzy control of the cleaning process and combining the data model with the operator’s experience, faults can be effectively avoided. Chen et al. (2014) designed a fault diagnosis system for combine harvesters based on a fuzzy neural network (FNN) algorithm, establishing a nonlinear mapping relationship between fault symptoms and fault types, processing input component speed values, and outputting fault diagnosis results. Jack and Nandi (2001) and Samanta et al. (2003) successfully applied SVM for bearing fault detection and experimentally verified its high engineering application value in fault diagnosis. Diez-Olivan et al. (2018) proposed a new algorithm based on SVM to assess sensor health and demonstrated its effectiveness in analyzing the time-dependent trends of ship diesel engine faults.

Support Vector Machine (SVM) theory has a solid mathematical foundation and rigorous derivations. It can transform classification and regression problems into optimization problems with constraints, which are solved through various mathematical methods, optimizing its algorithm and improving computational speed and performance. SVM theory has achieved excellent results in condition monitoring and fault warning applications (Liu et al., 2013; Chen et al., 2019; Ding et al., 2022; Ahmad et al., 2021; Peng et al., 2021). However, existing fault diagnosis methods, particularly those based on traditional machine learning (such as basic SVM), often face challenges such as poor generalization in small-sample, high-dimensional data scenarios and difficulty in parameter tuning. Deep learning approaches (e.g., CNN, LSTM, and reinforcement learning) have shown promising results but typically require extensive datasets and higher computational resources, making them less practical for real-time onboard applications.

To address these limitations, this study aims to predict clogging fault risks and dynamically adjust operational speed, ensuring the stable, long-term operation of unmanned combine harvesters. Instead of pinpointing specific fault locations, the primary focus is on managing the overall clogging risk to improve harvesting efficiency and reliability. Specifically, we propose a harvesting speed control system that integrates multi-component slip rate monitoring, an improved particle swarm optimization (IPSO)-optimized SVM model for accurate fault state classification, and a fuzzy logic-based algorithm for adaptive speed control. By collecting real-time data from Hall-effect sensors installed on critical harvesting components and utilizing 4G communication with cloud platforms, the system quickly and effectively reduces clogging risks. This approach significantly enhances operational efficiency, reduces downtime, and holds great potential for practical implementation in intelligent and unmanned agricultural machinery, extending its applicability to diverse farming environments and various agricultural machinery models.

The main innovations and contributions of this research are as follows:

1. Multi-component signal fusion: By collecting rotational speed signals from key components such as the header auger, conveyor, threshing drum, auger, and vibration screen, the system integrates slip rate data to reflect potential clogging signs across different transmission chains, overcoming the limitations of traditional single-component load feedback.
2. Efficient fault warning: Utilizing IPSO-SVM to classify small sample, high-dimensional clogging fault patterns enables accurate fault warning. The optimized algorithm enhances the SVM kernel function parameters and penalty factors, improving warning accuracy and robustness. Field tests validate that the fault warning model can correctly identify more than 98% of fault states.
3. Automatic speed regulation: Using fuzzy control, the system integrates fault warning results and feeding rate estimates into speed adjustment rules to enable real-time closed-loop control of operational load. This reduces clogging risks while ensuring fieldwork efficiency. Field tests confirm that the system can reduce speed within 0.5–2 seconds after detecting minor clogging, effectively preventing the occurrence of complete blockage failures.

To better present the research approach and experimental results, the structure of this paper is organized as follows: Section 2: Introduces the harvester parameters, power transmission structure, fault warning speed control system’s hardware and software framework, the IPSO-SVM-based fault warning principle, and the fuzzy-based speed control principle. Section 3: Describes the experimental design and results of feeding rate calibration, fault warning model performance verification, and speed control experiments. Section 4: Summarizes the research findings and conclusions, and discusses potential directions for future research.

## 2 Materials and methods

### 2.1 Harvester parameters and common faults

This study is based on the World Ruilong 4LZ-5.0 combine harvester, with key parameters listed in **Table 1**. The selection of this specific harvester was based on its affordability, ease of availability for field testing, and its widespread use as a commercial model featuring standard components common in modern combine harvesters. Moreover, all key mechanical parts are easily accessible for sensor installation, facilitating the implementation of electronic monitoring systems. The harvester’s hydraulic speed transmission (HST) system also simplifies the integration of automated speed control mechanisms. Importantly, the proposed fault prediction and speed control system is not limited exclusively to this particular model; it can be readily adapted to other combine harvesters sharing similar mechanical and electronic architectures.

**Table 1:** The main parameters of the World Ruilong 4LZ-5.0.

Parameter	Value	Unit
Combine dimensions (length- width- height)	4960 - 3950 - 2830	mm
Combine weight	3000	kg
Engine power	75	kw
Gear shift method	3 speeds HST	–
Travel speeds (at rated engine speeds)	Low speed: 0 to 1	m/sec
	Standard: 0 to 1.5	
	High speed: 0 to 2	
Cut width	2200	mm
Feeding capacity	5	kg/s
Operational efficiency	0.68 – 0.85	ha/h
Grain tank capacity	1.5	m^3^

The harvester’s power consumption is primarily divided into two parts: the drive system and the operational system. The load of the drive system is mainly influenced by factors such as ground friction resistance, slope resistance, the internal friction of the transmission system, and the inertia forces during vehicle acceleration and deceleration. The operational system’s load is primarily affected by factors such as the crop feed rate per unit time, the composition of the crop, and the working conditions of the respective operational components. The real-time power consumption of the drive system can cause fluctuations in the maximum load of the operational system.

**Figure 1:**
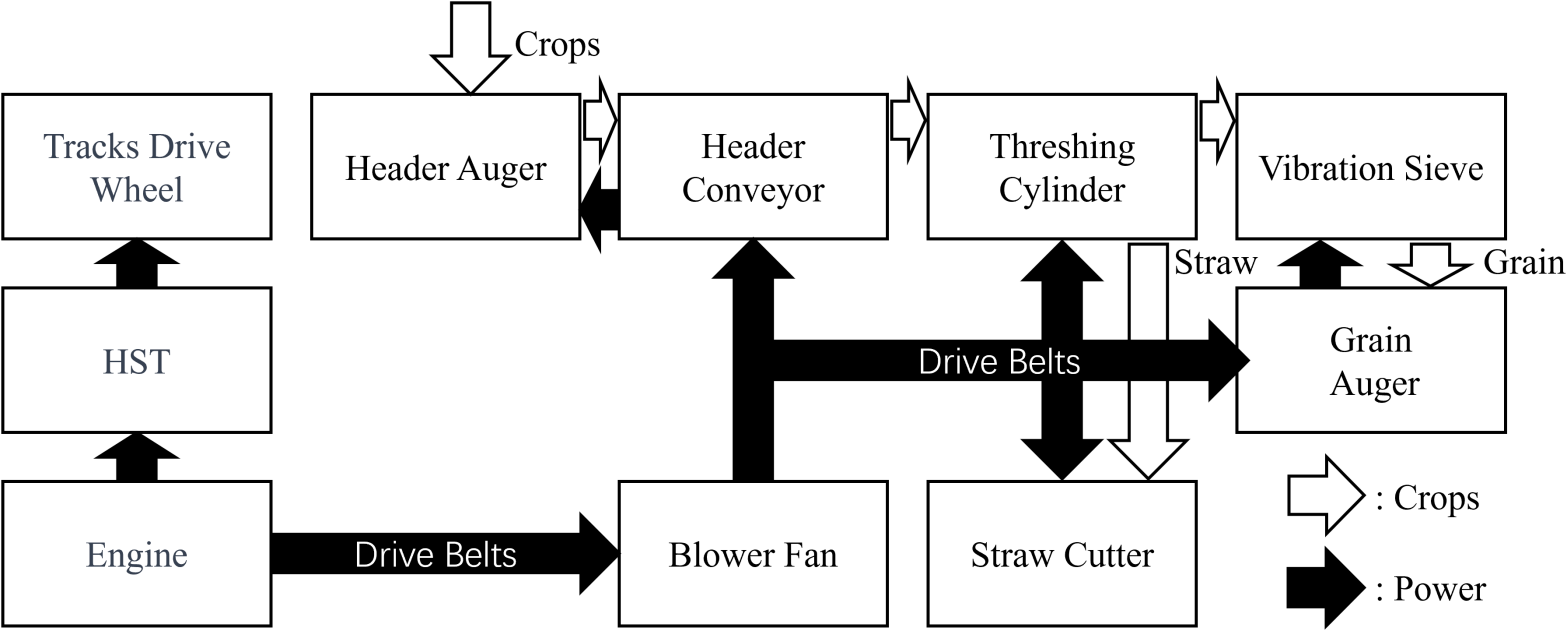
The power system structure of the 4LZ-5.0 combine harvester.

The crop transfer process and the power transmission process of the harvester are not consistent, which makes it challenging to infer crop clogging from monitoring the power system’s status. The crop transfer flow includes: cutter bar cutting the stems → feeding the crop into the header auger → conveyor chain rake → threshing cylinder → vibrating sieve and fan cleaning → secondary cleaning of residuals → grain is sent into the grain tank by the auger, while straw is sent to the straw cutter for grinding and discharge after passing through the threshing cylinder. The power transmission flow in the operational system includes: engine → fan →header conveyor→ header auger → cutter bar and reel, fan → threshing cylinder, fan → straw cutter, fan → grain auger → vibrating sieve, as shown in **Figure 1**.

**Figure 2:**
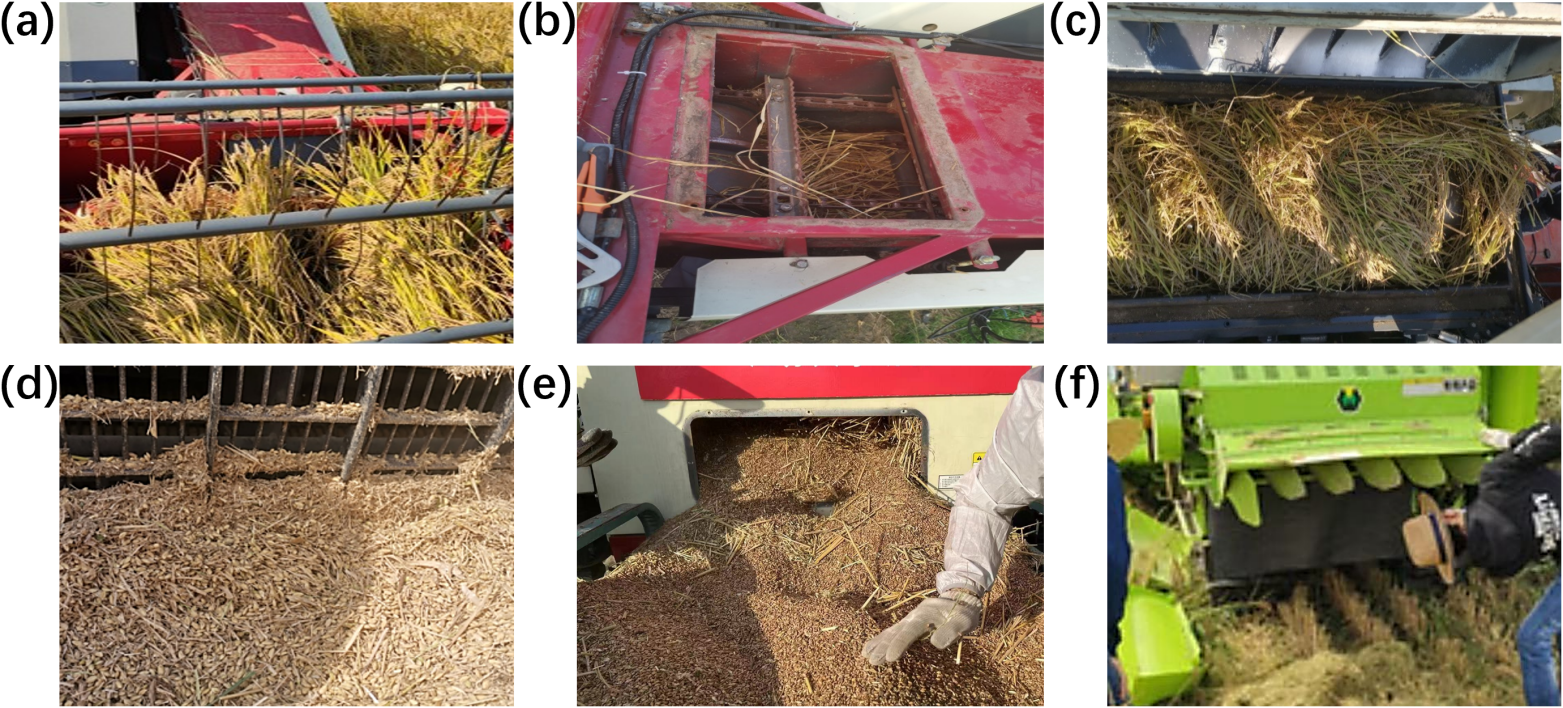
Common faults in combine harvesters. **(a)** Header auger clogging; **(b)** Header conveyor clogging; **(c)** Threshing cylinder clogging; **(d)** Vibrating sieve clogging; **(e)** Grain auger clogging; (f) Straw cutter clogging.

According to recent statistics on warranty and fault repair data (Chen et al., 2023), most mechanical clogs occur when key components in the transmission chain (such as the Header Auger, Conveyor, Threshing Cylinder, Grain Auger, Vibration Sieve, Straw Cutter, etc.) become obstructed or fail to operate smoothly due to crop accumulation or excessive load, as shown in **Figure 2**. The Blower Fan often serves as the driving axis for the transmission system, with most components maintaining a fixed speed ratio with the Blower Fan (such as the Header Auger and Vibration Sieve, which maintain a fixed speed ratio with their respective upstream components). When any component experiences clogging or slipping, its slip rate deviates significantly. Therefore, in this study, the slip rate of the pulley is used as the primary monitoring parameter, supplemented by monitoring the harvester’s forward speed and GPS positioning information to assess the feeding rate. An intelligent algorithm is then applied to comprehensively determine the fault risk value of the harvesting operation.

### 2.2 Overall design of the system

**Figure 3:**
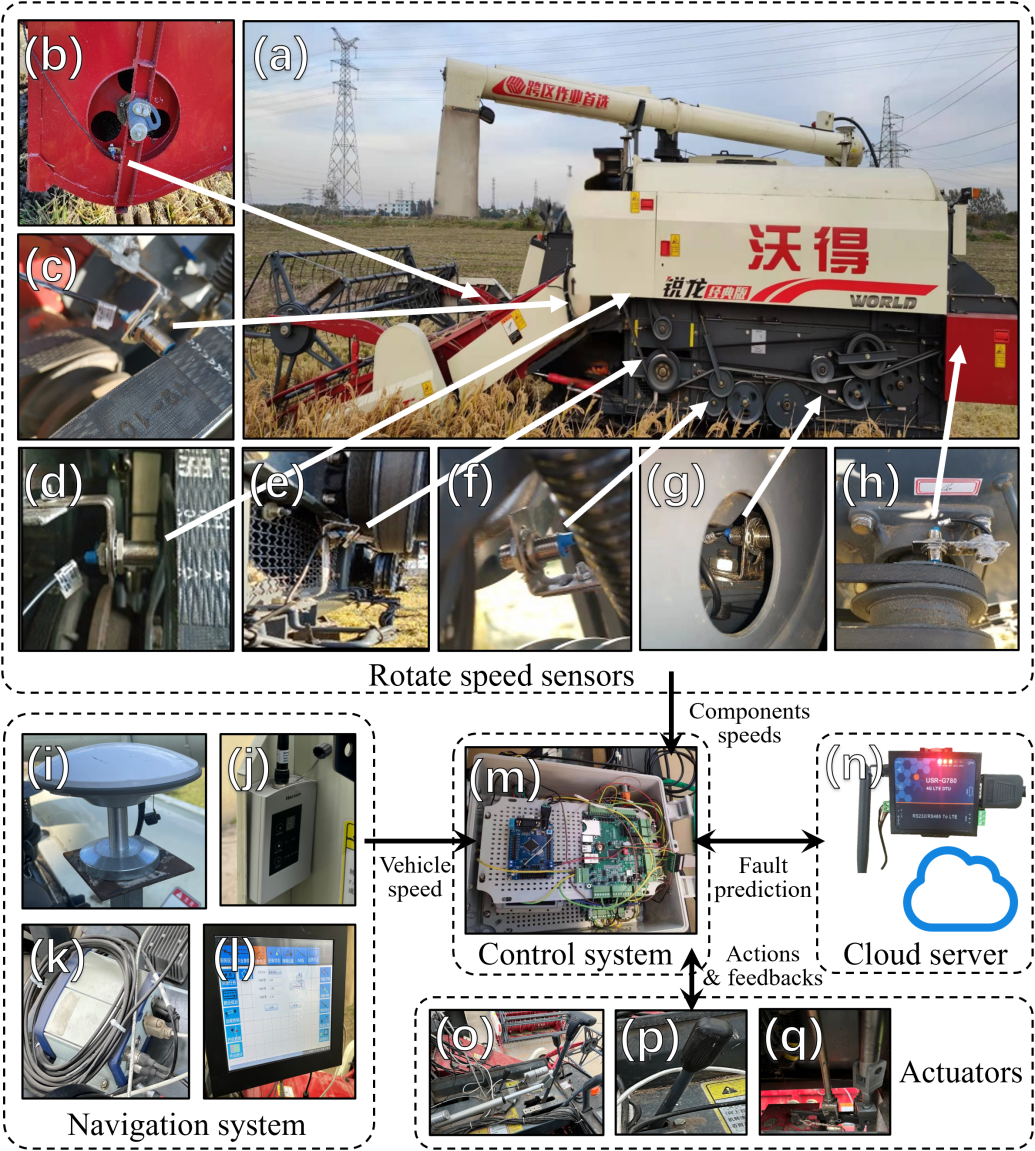
Hardware structure of fault warning and speed control system. **(a)** Combine harvester; **(b)** Header auger; **(c)** Header conveyor; **(d)** Threshing cylinder; **(e)** Blower fan; **(f)** Vibration sieve; **(g)** Grain auger; **(h)** Straw cutter; **(i)** Positioning antenna; **(j)** RTK radio; **(k)** GNSS receiver; **(l)** Navigation software; **(m)** Embedded Controller; **(n)** LTE-QTU Module; **(o)** HST Rod; **(p)** Accelerator; **(q)** Header lift rod.

To achieve fault warning and speed control during the operation of the combine harvester, the onboard hardware system used in this study is configured as shown in **Figure 3**, which includes:

#### 2.2.1 Operational component speed monitoring system

HR-M1850 Hall effect speed sensors (Omron (China) Co., Ltd.) are used to collect real-time data on the rotational speed of the drive pulleys for seven key operational components: Header Auger, Header Conveyor, Threshing Cylinder, Blower Fan, Grain Auger, Vibration Sieve, and Straw Cutter.

#### 2.2.2 Navigation system

The system, based on the UB482 positioning core board (Beijing Unicore Communications Co., Ltd.), is utilized for the harvester’s unmanned driving functionality and simultaneously collects vehicle speed information (Sun et al., 2022).

#### 2.2.3 Control system

The system, based on the EMB8600I embedded industrial control board (Beijing EmbedArm Co., Ltd.), processes sensor data, transmits it to the remote communication module, receives fault warning results from the cloud, and controls the vehicle’s actions.

#### 2.2.4 Remote communication system

The system, based on the G780 4G LTE DTU module (Jinan Youren Network Co., Ltd.), enables remote communication with the cloud server.

#### 2.2.5 Onboard actuator system

This includes the electrification of the HST speed change actuator and the Header lift actuator using electric push rods (YNT03, Nanjing Yongnuo Transmission Equipment Co., Ltd.), as well as the conversion of the original manual throttle to electric control via the embedded control board’s ADC interface.

**Figure 4:**
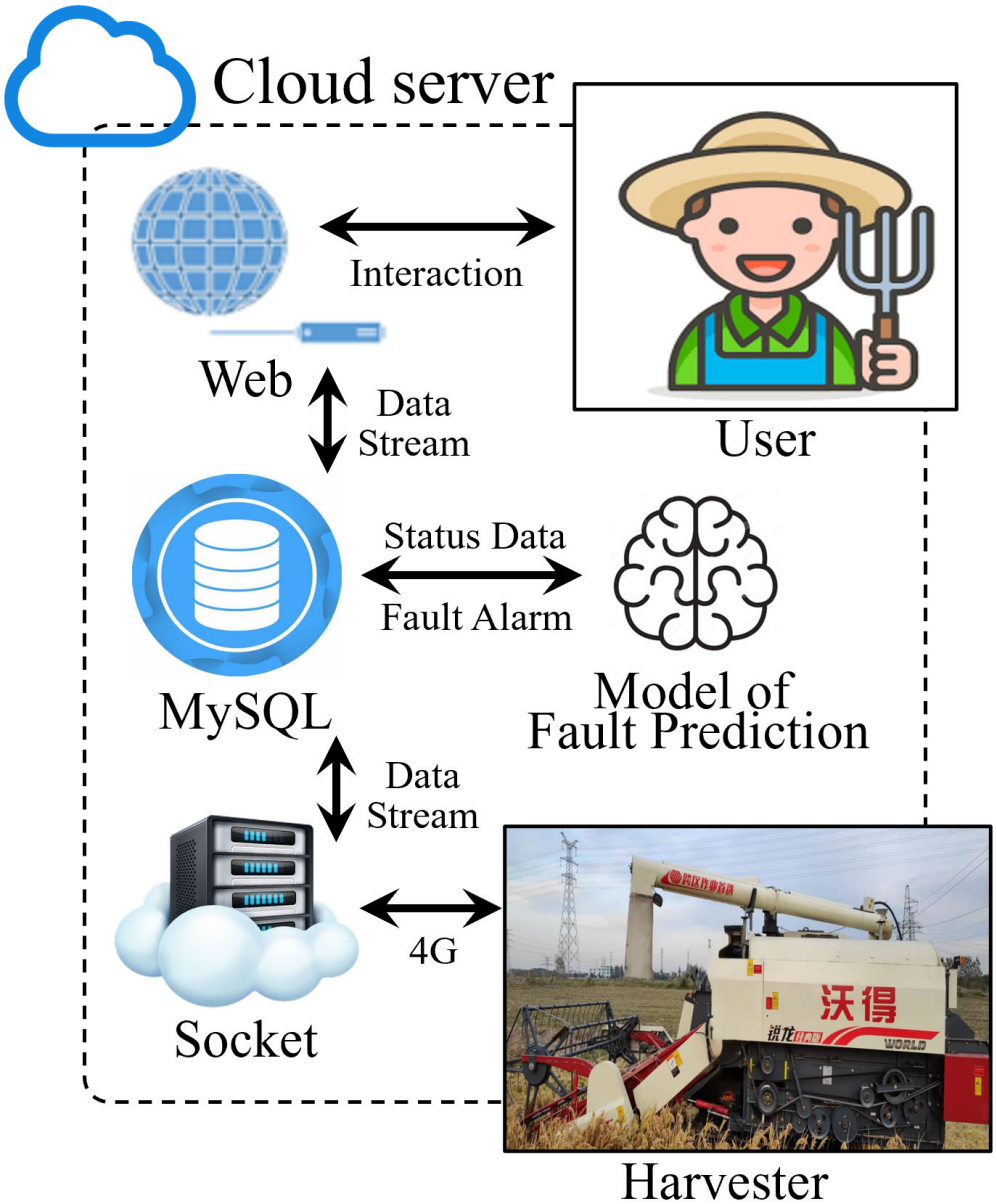
Cloud server architecture diagram.

The cloud server used in this study consists of a Socket server, database module, and web interface, as shown in **Figure 4**. The Socket server primarily handles communication with the onboard terminal, providing services such as data reception, information feedback, data protocol parsing, and fault warnings. The MySQL database module is responsible for the persistent storage of data received from the Socket server, fault codes generated by fault warnings, and other relevant information from the web service. The web interface handles remote monitoring of the harvester and human-machine interaction features (Zang et al., 2022).

The server is hosted on Alibaba Cloud ECS (Elastic Compute Service) and runs Windows Server 2008, ensuring stability and security. Communication between the system components is based on a TCP transparent transmission Socket protocol, with custom message formats for uploading sensor data and sending back fault warning states.

The development of the machine learning-based fault warning model for the combine harvester is primarily carried out using Python 3.6. The Scikit-learn library is used to build an IPSO-SVM classification model, which is trained using historical clogging fault data from the combine harvester. The trained model is serialized and saved using the pickle module. The Socket server loads the model file into memory and uses real-time rotational speed data to generate fault warning results.

### 2.3 IPSO-SVM-based fault risk warning model

The clogging fault in combine harvesters can be treated as a pattern recognition problem, with mainstream machine learning methods such as neural networks and Support Vector Machines (SVM) commonly applied. However, traditional SVM methods are sensitive to hyperparameter tuning, which significantly affects their generalization performance, especially under conditions with limited training samples. Similarly, particle swarm optimization (PSO)-based approaches can suffer from premature convergence, limiting their ability to find optimal solutions efficiently.

To overcome these limitations, this study adopts an improved particle swarm optimization (IPSO) strategy to enhance the generalization capability and accuracy of the SVM model. Specifically, the IPSO algorithm effectively optimizes the kernel parameters and penalty factors (*C* and σ) of the SVM, enabling better fault state classification performance. By introducing nonlinear transformations, the IPSO-SVM framework maps low-dimensional, non-separable data into high-dimensional space, enhancing fault prediction accuracy and robustness.

**Figure 5:**
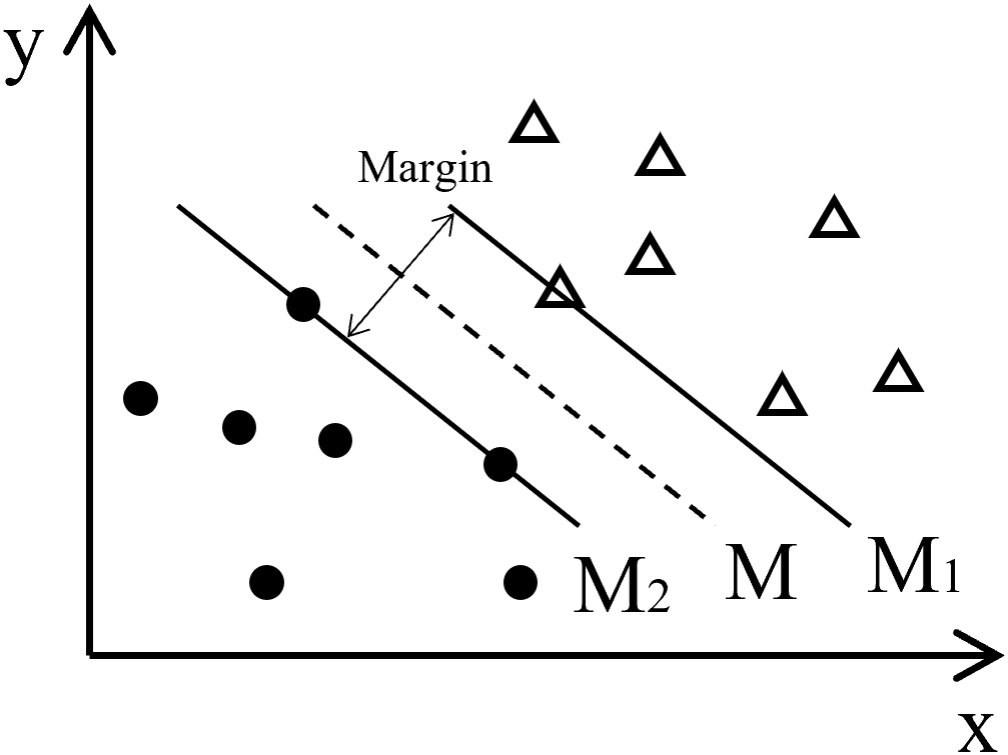
Schematic diagram of 2D linear separation.

SVM is a supervised learning method based on the maximum margin strategy. It classifies data in high-dimensional space by constructing an optimal hyperplane. As shown in **Figure 5**, in a two-dimensional space, suppose there are two types of samples, and linear classification can be achieved through multiple boundary functions (M). The optimal boundary is defined by support vectors. Support vectors (M1, M2) are the samples closest to the boundary, and the distance between them is the margin. Maximizing the margin helps enhance the model’s generalization ability, thus improving classification accuracy.

When this theory is extended to high-dimensional data, the linear decision boundary becomes a hyperplane. The goal is to determine the optimal hyperplane that accurately separates samples into their respective categories. To achieve this, the radial basis function (RBF) kernel is employed to transform nonlinear, non-separable input data into a higher-dimensional feature space, enabling linear separability. This transformation allows the SVM model to effectively classify fault states. Fault state classification follows a similar principle to the two-dimensional case illustrated in **Figure 5**, where the optimal hyperplane is defined by support vectors, clearly distinguishing normal and faulty states. The decision boundary generated by these support vectors divides the feature space into distinct classification regions. Samples that fall within each region are classified accordingly, based on their proximity to historical support vectors representing different fault states (e.g., normal, mild clogging, severe clogging, and complete blockage). This visual representation provides an intuitive understanding of the model’s classification performance and highlights how the SVM differentiates between normal and faulty conditions using the optimized hyperplane.

Let the dataset be $\text{T}=\{(x_{1},y_{1}),(x_{2},y_{2}),\cdots ,(x_{n},y_{n})\}$ , where $x_{i}\in R^{n}$ and $y_{i}\in \{1,-1\}$ , $x_{i}$ represents the input sample vector, and $y_{i}$ represents the sample label. The hyperplane can be represented by the following mathematical model (Equation 1):


(1) $$\omega^{T}x+b=0$$

where $\omega =(\omega_{1};\omega_{2};\cdots ;\omega_{n})$ is the normal vector of the hyperplane, and *b* is the offset, which determines the distance from the hyperplane to the origin. The distance *d* from a sample point to the hyperplane can be expressed as (Equation 2):


(2) $$d=\frac{\left|\omega^{T}x+b\right|}{∥\omega ∥}$$

When the hyperplane correctly classifies the samples, it should satisfy the following constraints (Equation 3):


(3) $$\left\{ \begin{array}{c} \omega^{T}x_{i}+b-1\geq 0,y_{i}=+1\\ \omega^{T}x_{i}+b+1\leq 0,y_{i}=-1 \end{array}\right.$$

Support vectors are the samples closest to the hyperplane, and the distance between the support vectors of the two classes is called the margin. This margin *D* can be expressed as (Equation 4):


(4) $$D=\frac{2}{∥\omega ∥}$$

The optimal hyperplane maximizes the margin, which is equivalent to minimizing (Equation 5):


(5) $$\underset{\omega ,b}{\min}\frac{∥\omega {∥}^{2}}{2}\;s.t.\;(\omega^{T}x_{i}+b)y_{i}\geq 1$$

By introducing Lagrange multipliers $\alpha_{i}$ (Rockafellar, 1993), the optimization problem can be transformed into the following form (Equation 6):


(6) $$L(\omega ,b,\alpha )=\frac{∥\omega {∥}^{2}}{2}-\sum_{i=1}^{n}\alpha_{i}((\omega^{T}x_{i}+b)y_{i}-1)$$

Taking partial derivatives of this Lagrange function and setting them to zero, we obtain (Equations 7, 8):


(7) $$\omega =\sum_{i=1}^{n}\alpha_{i}x_{i}y_{i}$$

(8) $$\sum_{i=1}^{n}\alpha_{i}y_{i}=0$$

Substituting the above into the Lagrange function transforms the problem into its dual form (Shawe-Taylor and Sun, 2011) (Equation 9):


(9) $$\begin{array}{c} \underset{\;}{\max}L(\alpha )=\sum_{i=1}^{n}\alpha_{i}\\ -\frac{1}{2}\sum_{i=1}^{n}\sum_{j=1}^{n}\alpha_{i}\alpha_{j}y_{i}y_{j}({\text{x}}_{\text{i}},{\text{x}}_{j})\;s.t.\{\begin{array}{c} \sum_{i=1}^{n}\alpha_{i}y_{i}=0\\ \alpha_{i}\geq 0 \end{array} \end{array}$$

This is solved using the Sequential Minimal Optimization (SMO) algorithm (Platt, 1998).

For non-linear separable problems, such as the relationship between rotational speed and slip rate data for fault risk in combine harvester failures, we introduce a nonlinear transformation. This maps low-dimensional, non-separable data into a high-dimensional space, making it separable. Using a kernel function, we can compute the inner product in the original space, avoiding direct computation in the high-dimensional space. Common kernel functions include the Radial Basis Function (RBF), polynomial kernel, and Sigmoid kernel (Hofmann et al., 2008). The RBF kernel is expressed as (Equation 10):


(10) $$k(x_{i},x_{j})=e^{-\frac{∥x_{i}-x_{j}{∥}^{2}}{\sigma^{2}}}$$

where σ is a parameter that significantly affects the SVM model’s performance, and it must be tuned to optimize the model.

To address the impact of measurement errors and noise on hyperplane optimization, we employ a soft margin method (Chen et al., 2004), optimizing by introducing slack variables ξ and a penalty factor *C*. The penalty factor determines the model’s tolerance for misclassification and affects the generalization and fitting capacity of the model.

The optimization problem for the optimal hyperplane can now be expressed as (Equation 11):


(11) $$\underset{\omega ,b,C}{\min}\frac{∥\omega {∥}^{2}}{2}+C\sum_{i=1}^{n}\xi_{i}\;s.t.\;(\omega^{T}\varphi (x_{i})+b)y_{i}\geq 1-\xi_{i}$$

The dual form of the optimization problem is (Equation 12):


(12) $$\begin{array}{c} \max L(\alpha )=\sum_{i=1}^{n}\alpha_{i}\\ -\frac{1}{2}\sum_{i=1}^{n}\sum_{j=1}^{n}\alpha_{i}\alpha_{j}y_{i}y_{j}k(x_{i},x_{j})\text{ s}.\text{t}.\text{ \{}\begin{array}{c} \sum_{i=1}^{n}\alpha_{i}y_{i}=0\\ 0\leq \alpha_{i}\leq C \end{array} \end{array}$$

Finally, by solving the dual problem of the Lagrange function, we obtain the decision function for the nonlinear SVM model (Equation 13):


(13) $$f(x)=\text{sign}(\sum_{i=1}^{n}k(x_{i},x)\alpha_{i}y_{i}+b)$$

To improve the classification performance of the SVM model, we adopted an improved particle swarm optimization (IPSO) algorithm to optimize SVM parameters C and σ . Traditional SVM models are sensitive to hyperparameter tuning, significantly affecting their generalization performance, especially with limited data. Conventional PSO often suffers from premature convergence, limiting the quality of parameter optimization. Therefore, the IPSO approach, featuring enhanced inertia weight adaptation, was used to accelerate convergence and improve optimization efficiency.

The position and velocity update rules for a particle in PSO are defined as follows (Equations 14, 15) (Cheng and Jin, 2015):


(14) $$V_{i}^{t+1}=\omega V_{i}^{t}+c_{1}rand_{\left[0,1\right]}(pbest_{i}-X_{i}^{t})+c_{2}rand_{\left[0,1\right]}(gbest-X_{i}^{t})$$

(15) $$X_{i}^{t+1}=X_{i}^{t}+V_{i}^{t+1}$$

where $V_{i}^{t}$ is the particle’s velocity vector, ω is the inertia weight, which controls the balance between global and local search, $c_{1}$ and $c_{2}$ are the cognitive and social learning factors, and rand_[0,1]_ is a random value between 0 and 1, adding randomness to the search process. $pbest_{i}$ is the best solution found by the particle, and gbest is the best solution found by the entire swarm. $X_{i}^{t}$ is the particle’s position at time *t*, representing the current state in the solution space.

During PSO, dynamic adjustments to the inertia weight ω and the learning factors $c_{1}$ and $c_{2}$ help accelerate convergence and avoid local optima. By adjusting these parameters, PSO effectively optimizes the SVM model parameters, improving classification accuracy. To address issues like premature convergence in traditional PSO (Nakisa et al., 2014), we propose a dual-improvement strategy:

1. A linear differential decrement strategy to adjust the inertia weight (Equation 16):

(16) $$\omega =(\omega_{s}-\omega_{e})\times {\left(\frac{k}{k_{max}}\right)}^{2}+(\omega_{e}-\omega_{s})\times (\frac{2\times k}{k_{max}})+\omega_{s}$$

where k is the current iteration, $k_{max}$ is the maximum number of iterations, $\omega_{s}$ is the initial inertia weight, and $\omega_{e}$ is the final inertia weight.

2. An asynchronous adjustment strategy to improve the size of the learning factors (Equations 17, 18):

(17) $$c_{1}=(\frac{C_{1s}+C_{1e}}{2})+(\frac{C_{1s}+C_{1e}}{2})\times cos(\frac{k\pi }{k_{max}})$$

(18) $$c_{2}=(\frac{C_{2e}+C_{2s}}{2})+(\frac{C_{2e}-C_{2s}}{2})\times cos(\frac{k\pi }{k_{max}})$$

where $C_{1s}$ and $C_{2s}$ are the initial individual and social learning factors, and $C_{1e}$ and $C_{2e}$ are the final individual and social learning factors.

In this study, the number of particles was set to 50, based on previous optimization studies that demonstrated this size effectively balances convergence speed and optimization quality. The maximum iterations (100) were empirically determined to ensure sufficient exploration without excessive computational cost (Liu et al., 2013; Chen et al., 2019; Ding et al., 2022; Ahmad et al., 2021).

Compared with conventional SVM and PSO-SVM models, the IPSO-SVM approach provides better classification results due to its efficient parameter optimization and improved convergence properties. The proposed approach thus effectively addresses clogging fault prediction challenges, ensuring long-term stable operation of unmanned harvesters.

The process of establishing the harvester clogging fault diagnosis model can be summarized as follows:

1. Collect data under normal, mild clogging, severe clogging, and complete blockage conditions through the experimental platform, extract slip rate features, normalize the data, and split the samples into a training set and a test set with a 4:1 ratio.
2. Use the IPSO algorithm in MATLAB 2022a to efficiently search for optimal SVM parameters, specifically kernel parameters and penalty factors.
3. Construct the IPSO-optimized SVM model in Python using the Scikit-learn library, perform offline training, and generate the fault warning model.
4. Deploy the trained IPSO-SVM model via the Socket server, uploading rotational speed data from the onboard DTU device to obtain fault prediction states in real time.

### 2.4 Fuzzy-based operation speed control system

Under the premise of properly adjusting the mechanical structure, clogging failures often occur during field operations of combine harvesters due to excessive feeding rates, which may result from factors such as high harvesting speeds, low cutting heights, high crop density, and excessive moisture content. During the initial stage of clogging, the issue can be effectively mitigated by appropriately reducing the vehicle’s speed. Skilled operators can often maximize harvesting speed while preventing clogging failures (Schwegman et al., 2021). Similarly, to ensure that unmanned combine harvesters can operate efficiently while avoiding clogging, they must be capable of automatically adjusting the harvesting speed based on variations in feeding rates and fault conditions. This process involves complex control challenges that are difficult to model precisely using traditional mathematical models.

Fuzzy control algorithms, which do not rely on exact mathematical models, are suitable for handling such imprecise control systems. By fuzzifying the fault states and feeding rates per unit time, and referencing manual operating experience, fuzzy control rules can be established. In this study, the fuzzy control rules were established based on extensive field trials and validated through operator feedback. By analyzing historical clogging events and operator interventions, optimal speed adjustments were determined for various clogging levels. This method ensures strong adaptability to real-world operational conditions. Subsequently, the harvester’s speed can be calculated and defuzzified. This strategy enables dynamic speed adjustment based on changes in fault status and feeding rates, thereby enhancing the adaptability of unmanned combine harvesters, reducing clogging, and improving operational efficiency.

Fuzzification of Input and Output Variables:

1. Fault status: The fault status level is output from the preliminary fault prediction model and corresponds directly to fuzzy quantization levels {Normal (N), Mild Clogging (LC), Severe Clogging (HC), Complete Blockage (CB)}.
2. Feeding intake (kg/s): The domain range is set to [0, 9] based on harvester parameters, with quantization levels {Small (S), Medium (M), Large (L)}, and a quantization factor of 3.
3. Harvesting speed (m/s): The domain range is set to [0, 1.2] based on harvester parameters, with quantization levels {Stop (ST), Slow (S), Medium (M), Fast (F)} and a quantization factor of 0.3. All are represented by Gaussian membership functions.
  Fuzzy Control Rule Design Principles:

1. Priority of fault state: In cases of severe clogging, the speed should be immediately reduced, or even stopped.
2. Feeding rate influence on speed: In the absence of faults, a higher feeding rate leads to a lower forward speed to prevent future clogging.
3. Adaptive adjustment: For mild clogging, speed can be slightly reduced to allow the system to recover; for severe clogging or complete blockage, speed should be significantly reduced or stopped. The fuzzy rules are shown in **Table 2**.

**Table 2:** Fuzzy control rule.

Fault status	Feeding Intake	Speed
N	S	F
N	M	M
N	L	S
LC	S	M
LC	M	S
LC	L	ST
HC	S	S
HC	M	ST
HC	L	ST
CB	Any	ST

Defuzzification Using the Centroid Method:

The centroid method is used for defuzzification, providing smooth output and is widely applied in industrial settings (Hung and Wu, 2002; Wu et al., 2019; Qi et al., 2022; Song et al., 2022; Zhang et al., 2024; Longaray et al., 2019; Pinochet et al., 2023). The calculation formula is as follows (Equation 19):


(19) $$V=\frac{\sum_{i=1}^{n}V_{i}f(V_{i})}{\sum_{i=1}^{n}f(V_{i})}$$

where *V* is the controller output, *n* is the number of rules, $f(V_{i})$ is the membership function, and $V_{i}$ is the corresponding speed value.

## 3 Experiments and discussion

### 3.1 Feed rate calibration experiment

The experimental field area is 6500 m², and the rice variety used is Nanjing 9108, with a crop-straw ratio of 1.65 and an overall moisture content of 40.4%. The air temperature ranged from 17-19°C. The experimental data were collected during the autumn harvest season in 2023. The harvesting operation was conducted at specific cutting widths and cutting heights to ensure consistency and comparability of the data. During the harvesting process at various locations in the experimental field, it was assumed that the crop’s growth density and moisture content were uniform. Therefore, the actual feeding rate could be calibrated by controlling the cutting width and cutting height. The formula for calculating the feeding rate is (Equation 20):


(20) Q=mS=mdv

where 𝑄 is the actual feeding rate of the harvester (kg/s), *m* is the crop mass per unit area (kg/m²), *S* is the crop area harvested per unit time (m²/s), *d* is the harvester’s cutting width (m), 𝑣 is the harvester’s operating speed (m/s).

**Figure 6:**
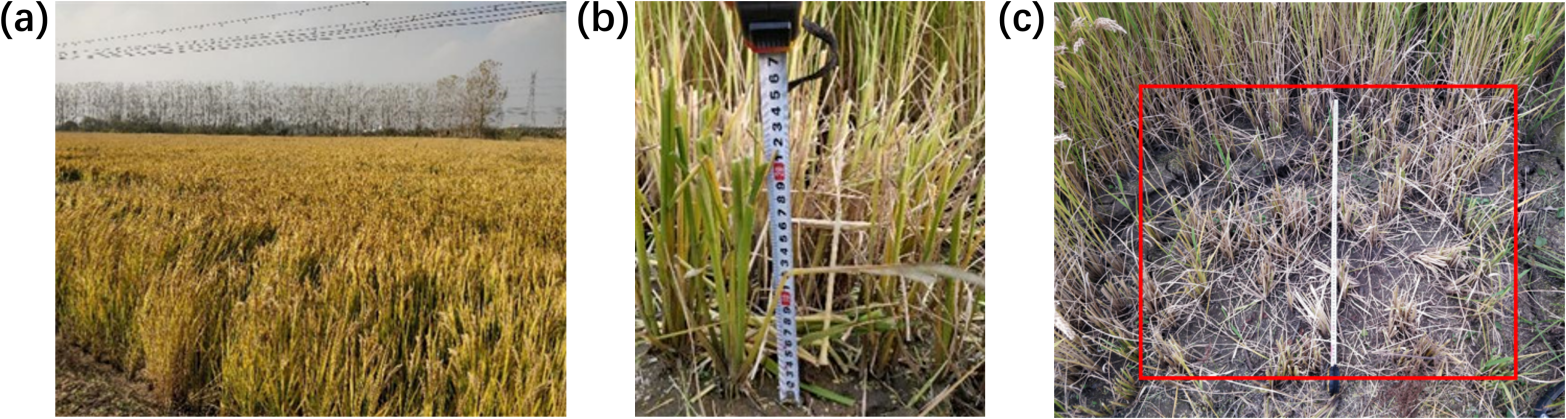
Feed rate calibration experiment. **(a)** General view of the experiment field; **(b)** Cutting height measurement; **(c)** Reaping area survey.

A crop block of approximately 4 square meters was selected to calibrate the crop mass per unit area, with cutting heights maintained at 0.15 m, 0.25 m, and 0.35 m. Manual harvesting was performed, and the harvested crop was weighed. The field conditions are shown in **Figure 6a**, the stubble conditions in **Figure 6b**, and the harvested area in **Figure 6c**. The weighing results for each crop block are presented in **Table 3**.

**Table 3:** Weighing results of crop blocks.

Crop number	Cutting height (m)	First weighing (kg)	Second weighing (kg)	Third weighing (kg)	Mass-area ratio (kg/m²)	Average mass-area ratio (kg/m²)
1	0.15	21.01	21.00	21.02	5.56	5.56
2		22.16	22.12	22.21	5.71	
3		21.22	21.20	21.22	5.60	
4		21.40	21.41	21.40	5.61	
5		21.07	21.10	21.09	5.57	
6		19.84	19.89	19.88	5.49	
7		21.43	21.42	21.41	5.62	
8	0.25	19.84	19.82	19.84	4.96	5.05
9		21.02	20.99	21.06	5.26	
10		20.28	20.25	20.27	5.07	
11		20.56	20.57	20.56	5.14	
12		20.13	20.16	20.15	5.04	
13		18.94	18.97	18.97	4.74	
14		20.68	20.68	20.66	5.17	
15	0.35	16.91	16.88	16.89	4.04	4.04
16		18.07	18.02	18.11	4.06	
17		17.28	17.25	17.27	4.05	
18		17.66	17.68	17.67	4.06	
19		17.02	17.04	17.03	4.04	
20		15.94	15.97	15.96	4.00	
21		17.22	17.21	17.20	4.05	

A quadratic function was used to fit the experimental data, yielding the cutting height-feeding rate relationship for the current field was calculated as Equation 21:


(21) $$Q=(-25h^{2}+4.9h+5.39)dv$$

where *h* is the cutting height (m).

It can be inferred from the above equation that, with constant cutting height and cutting width, the harvester’s speed is linearly related to the feeding rate.

By fixing the crop variety, crop-straw ratio, and overall moisture content, the feeding rate calibration experiment in the field was completed. This provides data on the feeding rate conditions for harvesting fields under similar conditions, supporting the subsequent development of speed control strategies to prevent clogging. However, the actual harvesting feeding rate is influenced by multiple factors, such as crop variety, crop-straw ratio, overall moisture content, growth density, lodging condition, and the harvester’s technical state (maintenance condition, wear degree, power configuration). Real-time calculation of the harvesting feeding rate is beyond the scope of this study. Based on the weighing results, it was observed that the growth density of the rice in the experimental field was relatively uniform, with minimal variation in crop mass per unit area. Therefore, this study only establishes the cutting height-feeding rate relationship through the equation above.

### 3.2 Performance verification of fault prediction model

To validate the practical application of the IPSO-SVM-based clogging fault warning model in field environments, this study involved speed state data collection from the pulleys of seven key components: Blower Fan, Header Auger, Header Conveyor, Threshing Cylinder, Vibration Sieve, Grain Auger, and Straw Cutter. The study included offline model training and testing, as well as online fault warning experiments. The model inputs consisted of the slip rate features from seven channels, while the output was the fault warning state labels, with a corresponding relationship between state labels and fault types as shown in **Table 4**. The normalization formula for the pulley slip rate features is (Equation 22):

**Table 4:** Harvester status categories and classification labels.

Status category	Classification label
Normal Lightly clogging	1 2
Heavily clogging	3
Completely blocked	4


(22) $$x^{,}=1-\frac{x-x_{min}}{x_{max}-x_{min}}$$

**Figure 7:**
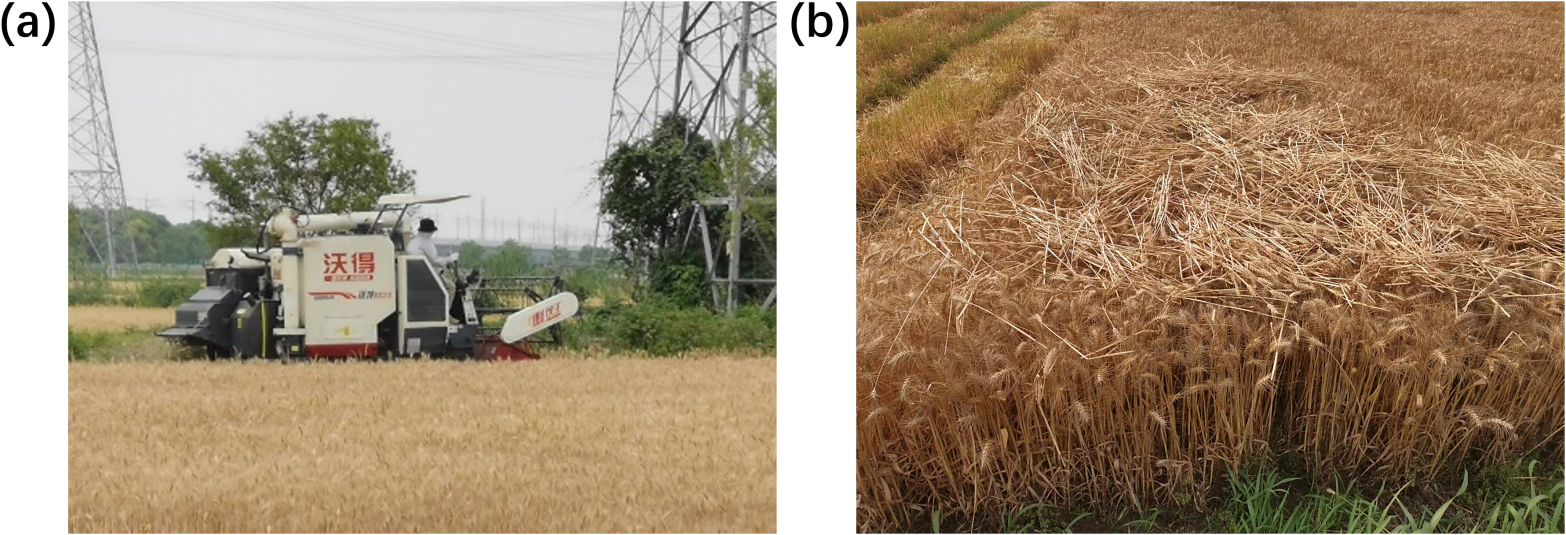
Fault Setting experiment. **(a)** Lower cutting table and increase speed; **(b)** Increase crop.

**Figure 8:**
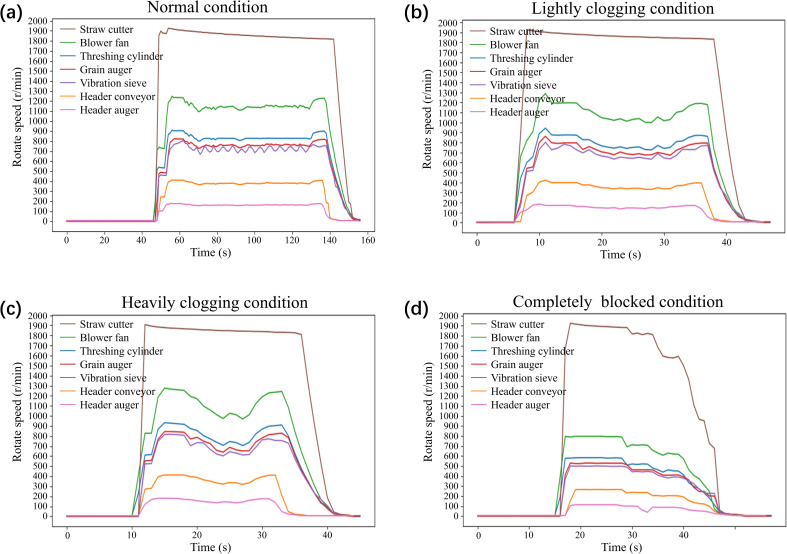
Speed data of each part. **(a)** Rotate speed in normal condition; **(b)** Rotate speed in lightly clogging condition; **(c)** Rotate speed in heavily clogging condition; **(d)** Rotate speed in completely blocked condition.

The experimental field was planted with the rice variety Nanjing 9108, with an average plant height of approximately 0.95 meters and a moisture content of about 40.3%. The engine’s rated speed was set to 2500 r/min, and the cutting width was 2 meters. Based on typical clogging fault scenarios in real-world conditions, human interventions such as lowering the cutting height, increasing harvesting speed, and increasing crop density were used to induce faults. The field experiment setup is shown in **Figure 7**. The harvesting machine’s rotational speed data under various fault states is presented in **Figure 8**. After excluding outliers and missing values, a total of 1071 valid data points were obtained. The fault warning classification labels were manually annotated, with 267 instances of normal state, 267 instances of mild clogging, 267 instances of severe clogging, and 270 instances of complete blockage. The training and testing datasets were randomly divided in a 4:1 ratio from each category.

**Figure 9:**
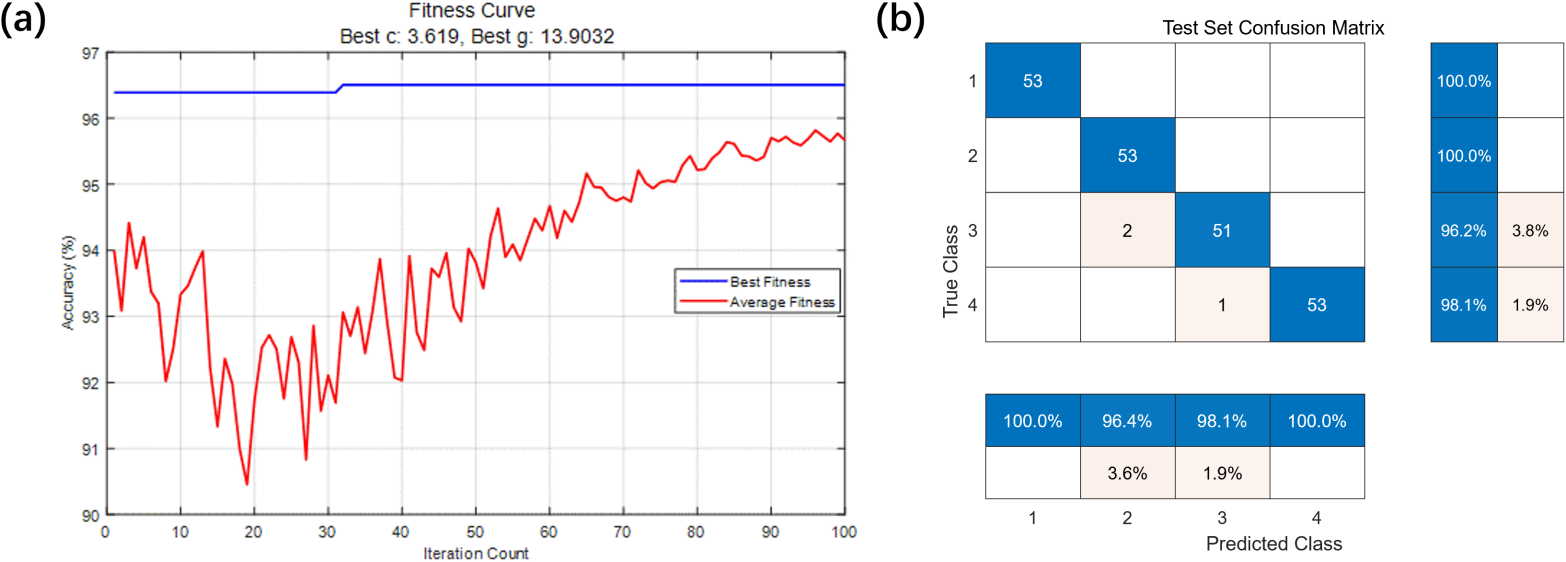
Fault Setting experiment. **(a)** Fitness change curve; **(b)** IPSO-SVM test set confusion matrix.

The IPSO-SVM model was built using MATLAB 2022a, where the IPSO population size was set to 50, the maximum number of iterations $k_{max}$ was set to 100, the initial inertia weight $\omega_{s}$ was set to 2, the final inertia weight $\omega_{e}$ was set to 0.4, and the learning factors were set as $C_{1s}$ =1.5, $C_{1e}$ =0.5, $C_{2s}$ =0.7 and $C_{2e}$ =2.0. The SVM kernel function used was the radial basis function (RBF). The feature data for both the training and testing datasets were normalized and input into the model for parameter optimization. The resulting fitness curve is shown in **Figure 9a**, where the optimal SVM parameters *C* and σ were found to be 3.6190 and 13.9032, respectively. The best fitness value of 96.50% was achieved at the 32nd generation. The testing dataset was then input into the trained model, and the confusion matrix is shown in **Figure 9b**. The overall classification accuracy of the model was 98.59%. The classification errors occurred because, during the initial stage of clogging, the difference in severity was not clearly reflected in the rotational speed data. When severe clogging occurred, the rotational speed data rapidly decreased. However, the sampling speed of the measurement system was limited and could not clearly distinguish between severe clogging and complete blockage.

Despite achieving high classification accuracy, the proposed system is subject to certain experimental errors and uncertainties. Three primary sources of error were identified: firstly, sensor inaccuracies in slip rate measurements, as sensor sensitivity and installation conditions might cause minor deviations in collected data; secondly, variability in environmental conditions, such as fluctuations in crop density, moisture content, and soil properties, which may impact model performance; and thirdly, potential delays in data transmission via the 4G communication module, resulting in slight discrepancies in fault detection timing and subsequent speed adjustments. To account for these uncertainties, confidence intervals have been incorporated into **Table 5**, providing a statistical measure of reliability when comparing the performance of different classification models. Confidence intervals for classification accuracy were computed based on the binomial normal approximation at a 95% confidence level (Brown et al., 2002), using the formula below (Equation 23):

**Table 5:** Comparison Table of Classification Accuracy of Different Models.

Status category	SVM (%)	PSO-SVM (%)	IPSO-SVM (%)
Normal	73.58 ± 5.30	86.79 ± 4.05 100.00 ± 1.12¹	100.00 ± 1.12¹ 100.00 ± 1.12¹
Lightly clogging	100.00 ± 1.12¹		
Heavily clogging	60.38 ± 5.86	88.68 ± 3.80	96.23 ± 2.27
Completely blocked	75.93 ± 5.10	85.19 ± 4.23	98.15 ± 1.61
Total	77.46 ± 2.51	90.15 ± 1.78	98.59 ± 0.71

1. For accuracies that are exactly 100.00%, the Rule of Three was applied, i.e., ±(3/n)×100%, to avoid a zero-width confidence interval under the normal approximation.


(23) $$\hat{p}\pm z_{\alpha /2}\sqrt{\hat{p}(1-\hat{p})/N}$$

where $\hat{p}$ is the sample accuracy, $z_{\alpha /2}$ is the z-value at the selected confidence level (for example, at 95% confidence, $z_{0.025}\approx 1.96$ ), and *N* is the test set size.

To evaluate the performance of the IPSO-SVM fault diagnosis model, a comparison was made with the PSO-SVM and traditional SVM models using the same training and testing datasets. The results, along with their corresponding 95% confidence intervals calculated using the normal approximation, are summarized in **Table 5**. The total accuracy of the IPSO-SVM model was 8.44% and 21.13% higher than that of the PSO-SVM and traditional SVM models, respectively. The inclusion of confidence intervals confirms the statistical reliability of these improvements. These results clearly demonstrate that the IPSO algorithm is more effective in optimizing the SVM parameters compared to the standard PSO algorithm, thus enhancing the generalization capability and classification accuracy of the SVM model.

Additionally, although the IPSO-SVM model demonstrated high accuracy, misclassification errors such as false positives and false negatives can still occur. False positives—incorrectly identifying normal states as fault conditions—might trigger unnecessary speed adjustments, potentially reducing operational efficiency. However, preventing fault escalation remains the primary goal; thus, the trade-off of occasionally reduced efficiency is considered acceptable. Conversely, false negatives—failing to detect actual faults—may lead to delayed corrective actions, but since faults typically worsen over time, subsequent detections are more likely to trigger accurate fault identification. Therefore, neither false positives nor false negatives significantly compromise the eventual execution of the fault-adjusted speed control system. Future research will explore strategies such as threshold optimization and ensemble learning techniques to minimize the impact of these misclassifications and further improve system reliability.

### 3.3 Operation speed control experiment

To validate the effectiveness of the cloud server and onboard control system’s fault warning-speed control system, this study conducted field tests for unmanned variable-speed rice harvesting. The experimental field was planted with the rice variety Nanjing 9108, with an average plant height of approximately 1.1 meters and an overall moisture content of 36.8%. The unmanned harvester’s automatic driving system is detailed in previous research (Zhang et al., 2023, 2025), and the onboard fault warning-speed control system continuously uploads pulley speed data from each component to the cloud server in real-time, while also receiving fault warning statuses from the cloud server. The embedded controller calculates the feeding rate per unit time based on the harvester’s real-time speed, assuming constant cutting height and cutting width. It then performs fuzzy logic operations to adjust the target speed based on the fault warning status and controls the HST speed adjustment actuator to regulate the vehicle’s speed.

**Figure 10:**
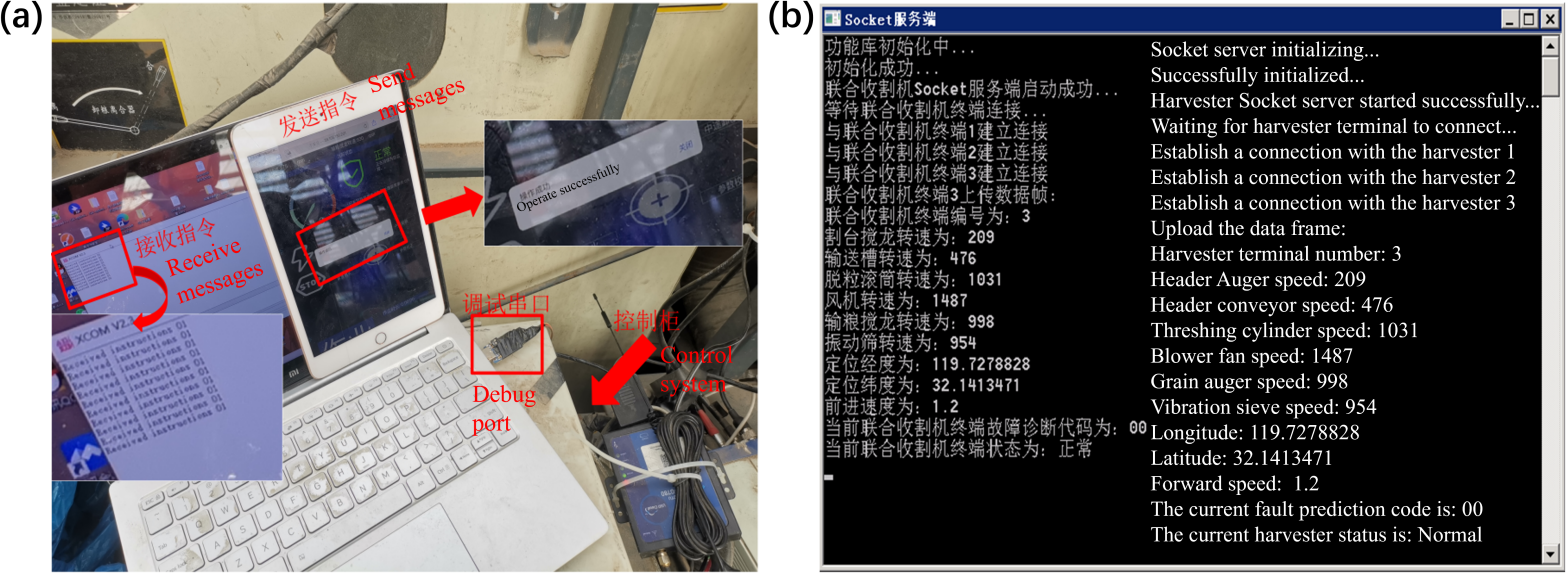
Cloud server communication experiment. **(a)** Onboard terminal tests; **(b)** Server communication window

**Figure 11:**
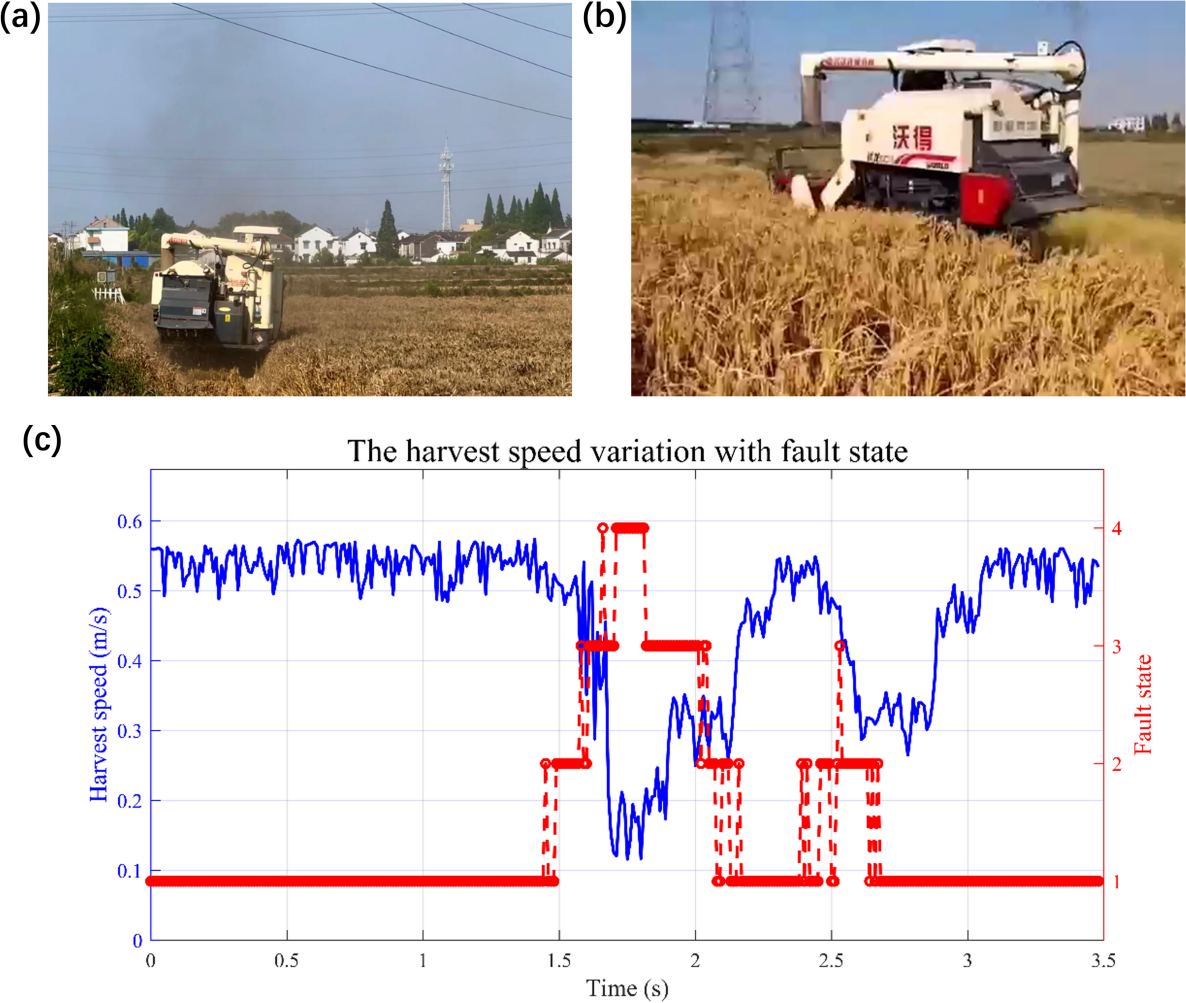
Fault prediction and speed regulation experiment. **(a)** Heavily clogging with black smoke caused by belt slippage; **(b)** Unmanned harvesting with speed regulation; **(c)** Plot of harvest speed variation with fault state.

After multiple field trials, the harvester equipped with the fault warning-speed control system did not experience complete blockage. It was able to reduce speed within 0.5–2 seconds during minor clogging events, effectively preventing fault escalation. Once the operational conditions were restored and the fault warning was cleared, the system promptly increased the speed to ensure operational efficiency. These results demonstrate that the system has good real-time responsiveness and robustness, and can effectively enhance the application value of unmanned combine harvesters. The communication process between the cloud server and the onboard terminal is shown in **Figure 10a**; the cloud server information window is shown in **Figure 10b**. The experimental simulation of heavily clogging accompanied by black smoke due to belt slippage is shown in **Figure 11a**; the unmanned harvester reducing speed and waiting for fault recovery is shown in **Figure 11b**; and the fault risk state and speed variation trend during the speed adjustment process is shown in **Figure 11c**.

Field tests confirm that this system can effectively prevent complete blockage in the harvester, ensuring long-term stable operation of the unmanned harvester. Moreover, this approach demonstrates promising potential for integration into smart farming platforms, enabling real-time monitoring and predictive maintenance to enhance overall agricultural management efficiency. The proposed system can be scaled effectively to multiple harvesting machines, supporting coordinated operation and grain discharge in cooperative fleets. Additionally, compatibility with emerging IoT and edge computing solutions can further improve real-time decision-making capabilities, expanding the broader impact of this technology in agriculture.

However, there are still some limitations:

1. The real-time calculation of feeding rate in this study relies on prior field calibration data, which is influenced by multiple factors such as overall moisture content, growth density, lodging condition, and the harvester’s technical state. Therefore, the application of this fault warning-speed control system requires reliable and user-friendly feeding rate prediction methods. Future research will focus on further exploration in this area.
2. The dataset used in this study comprises only 1,071 data points, which may not be sufficient to robustly validate the performance of a machine learning model. Moreover, these data were collected primarily from a single experimental field, potentially limiting the generalizability of the model to different environmental and operational conditions. Future research will aim to collect more extensive data from multiple fields and varied operational conditions to enhance the robustness and generalization of the model.
3. The IPSO-SVM-based fault warning model classifies fault states by analyzing the slip rates of multiple component pulleys, with manual labeling used for fault classification in the training set. While IPSO-SVM models offer better interpretability and are well-suited for small datasets, they rely on feature engineering and may struggle to capture complex, high-dimensional relationships as effectively as deep learning models (Chen et al., 2019; Ding et al., 2022; Li et al., 2025). Conversely, deep learning approaches, such as CNNs and LSTMs, excel in feature extraction but require large-scale training datasets and substantial computational power. Given the limited dataset size in this study, directly implementing deep learning models would pose challenges in both model training and generalization (He et al., 2023; Kakhi et al., 2024; Xiong et al., 2023). Moreover, deploying the IPSO-SVM model across different harvester types and enhancing fault classification accuracy would significantly increase the manual labeling workload. Future research will focus on developing automated and intelligent fault data labeling methods, as well as exploring hybrid approaches that integrate deep learning for feature extraction with SVM for classification, aiming to further improve model performance and adaptability.
4. The communication between the onboard system and the cloud server relies on the 4G DTU module, and its timeliness is significantly affected by on-site signal coverage. In areas without 4G signal availability, the transmission of remote monitoring commands and fault warning information will be delayed or lost, thereby compromising the real-time performance and reliability of the system. To address this limitation, future research will focus on deploying a simplified fault warning model directly on the onboard embedded system to ensure basic fault detection and speed control functions even under poor communication conditions.
5. Energy consumption considerations remain to be further evaluated. While the proposed system introduces minimal additional power demands due to the use of low-power embedded sensors, its adaptive speed control mechanism may contribute to improved fuel efficiency by reducing unnecessary high-speed operations. However, a more comprehensive real-time analysis of the harvester’s power consumption under different operating conditions is needed. Future research will focus on optimizing energy efficiency by integrating real-time power monitoring and adaptive energy management strategies.

## 4 Conclusion

To improve the stability of unmanned combine harvesters during long-term field operations and maintain harvesting speed and efficiency while avoiding clogging failures, this study developed a fault warning and speed control system based on the IPSO-SVM predictive model and Fuzzy control algorithm. The system was evaluated through extensive field trials, demonstrating its ability to effectively prevent complete clogging failures and ensure continuous operation. The main conclusions are as follows:

1. Clogging state prediction based on multi-component slip rate fusion: By monitoring the rotational speeds of key components such as the Blower Fan, Header Auger, Header Conveyor, Threshing Cylinder, Vibration Sieve, Grain Auger, and Straw Cutter, and extracting slip rate features, an IPSO-SVM model was developed that accurately identifies over 98.5% of fault states.
2. Feeding rate calibration and fuzzy speed control strategy: Field experiments demonstrated that the cutting height and crop mass per unit area have a quadratic relationship. This relationship, combined with real-time speed, can approximate the feeding rate. Based on the Fuzzy control strategy, the system intelligently adjusts the speed by considering both feeding rate and fault prediction states. The system successfully reacts within 0.5–2 seconds following minor clogging, preventing fault escalation and significantly reducing the occurrence of complete clogging failures.
3. Engineering application feasibility and future adaptability: The system’s reliability and scalability were validated in field environments by integrating the harvester’s electronic control modifications, onboard embedded controllers, 4G communication, and cloud servers. While the current study focused on a specific harvester model, future research will explore its adaptability across different harvester types to verify its broader applicability.
4. Future research directions: To further enhance the system’s performance, future work will focus on (1) testing on different harvester models to verify adaptability, (2) improving real-time processing capabilities for cloud-integrated control, and (3) integrating deep learning techniques to refine fault prediction accuracy. These advancements will contribute to optimizing fault prediction efficiency and ensuring more effective real-time operational adjustments.

## Data Availability

The original contributions presented in the study are included in the article/**Supplementary Material**. Further inquiries can be directed to the corresponding author.
