# Supplementary material for: Research on fault prediction and speed control system for unmanned combine harvesters based on IPSO-SVM and fuzzy logic
Source: Front Plant Sci. 2025 Jun 3;16:1577175. doi: 10.3389/fpls.2025.1577175 (PMC12170651; doi:10.3389/fpls.2025.1577175)
Supplement: Supplementary file 1 [file DataSheet1.docx]

Supplementary Material

# Supplementary Figures and Tables

## Supplementary Figures


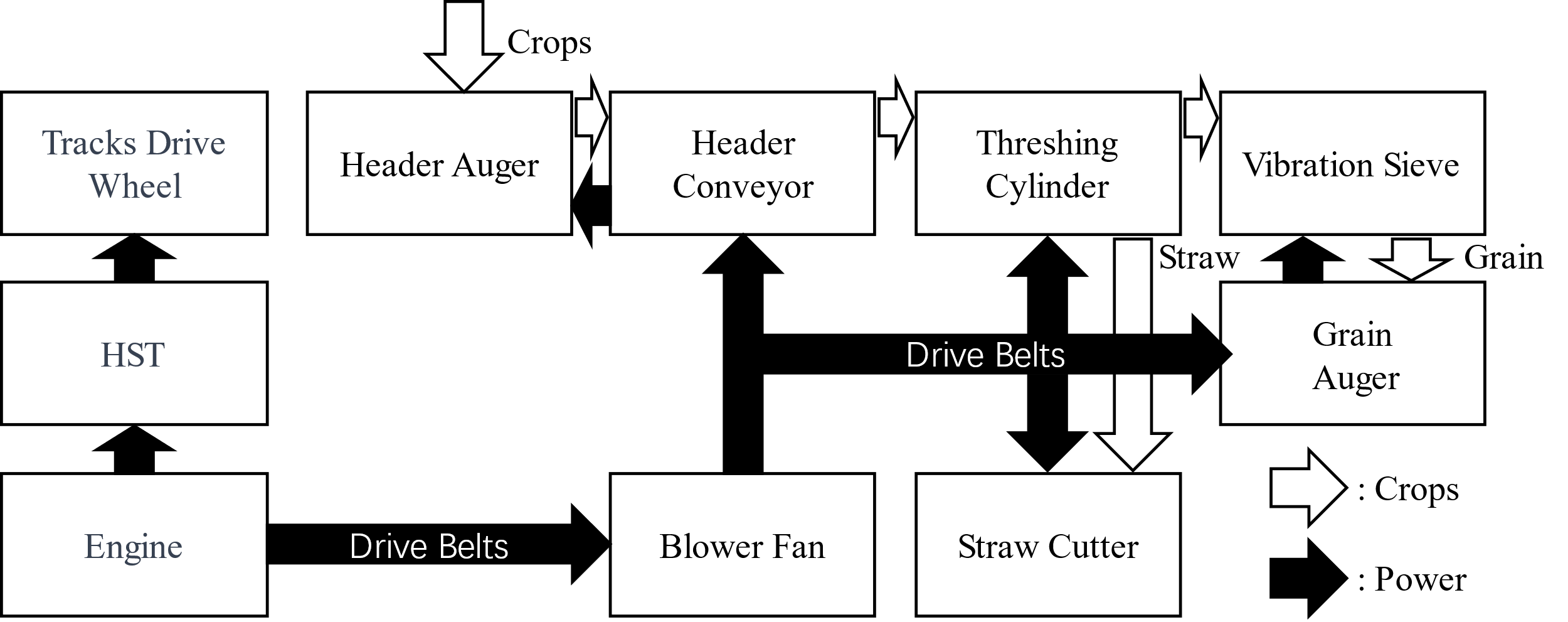


Figure 1. The power system structure of the 4LZ-5.0 combine harvester


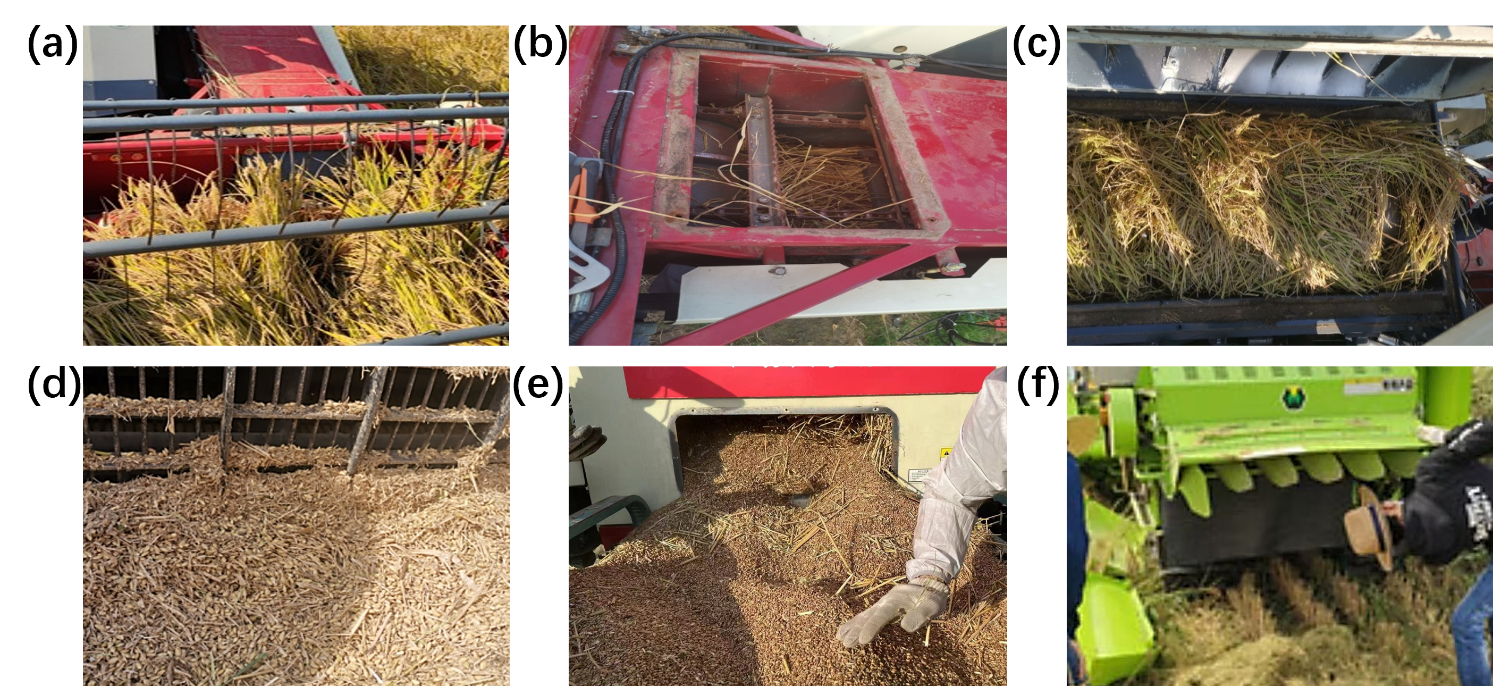


(a) Header auger clogging; (b) Header conveyor clogging; (c) Threshing cylinder clogging; (d) Vibrating sieve clogging; (e) Grain auger clogging; (f) Straw cutter clogging

Figure 2. Common faults in combine harvesters


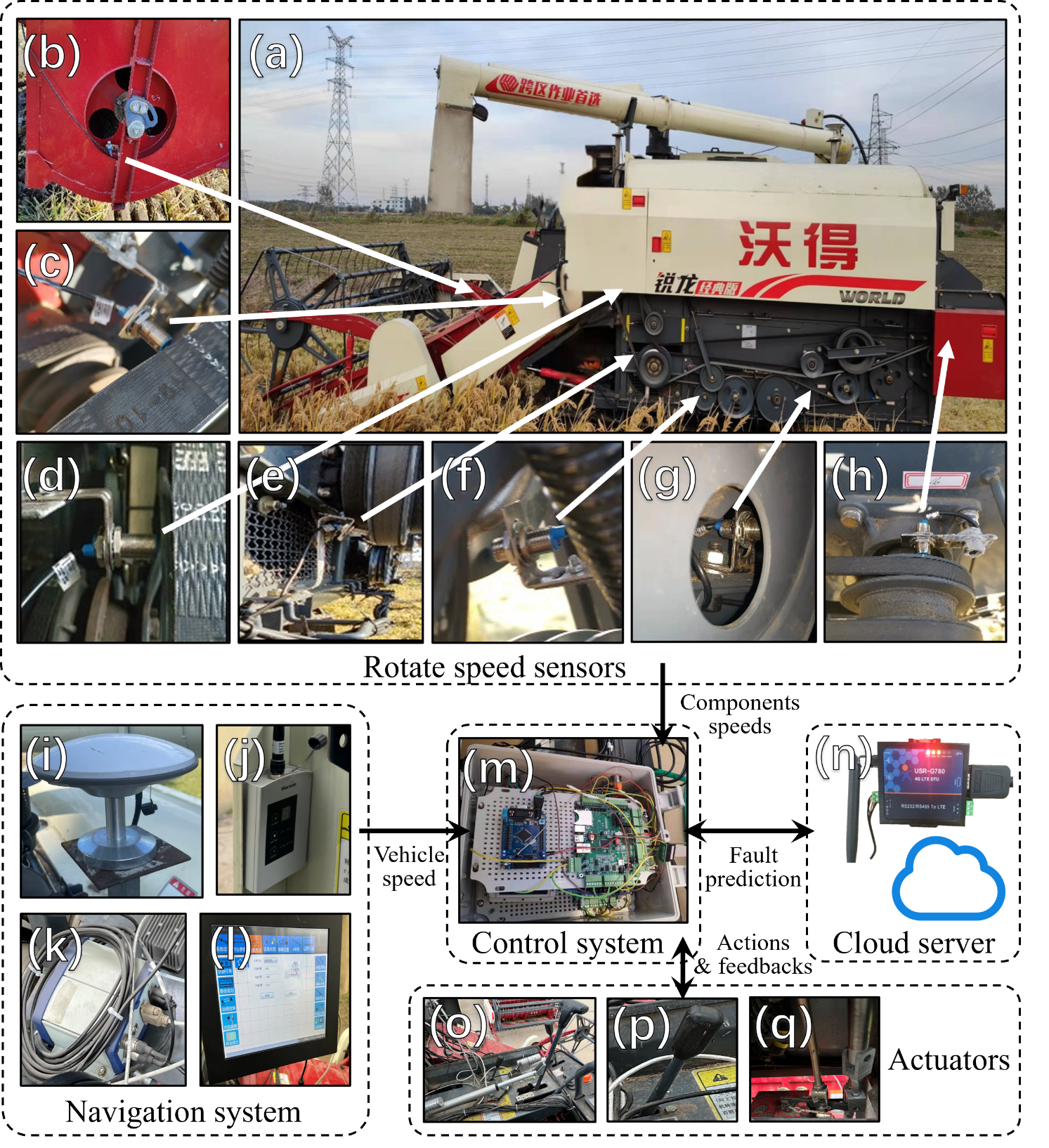


(a) Combine harvester; (b) Header auger; (c) Header conveyor; (d) Threshing cylinder; (e) Blower fan; (f) Vibration sieve; (g) Grain auger; (h) Straw cutter; (i) Positioning antenna; (j) RTK radio; (k) GNSS receiver; (l) Navigation software; (m) Embedded Controller; (n) LTE-QTU Module; (o) HST Rod; (p) Accelerator; (q) Header lift rod

Figure 3. Hardware structure of fault warning and speed control system


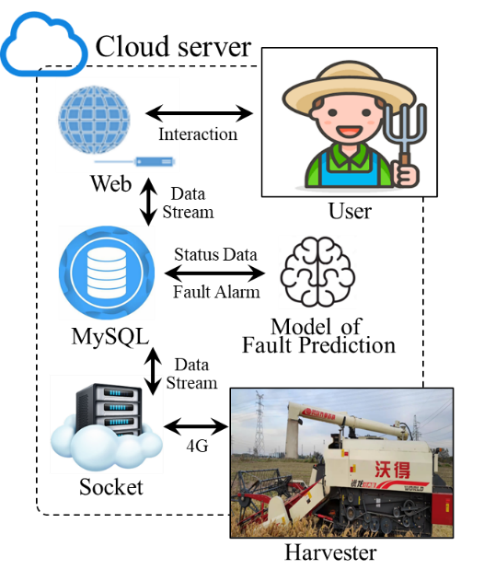


Figure 4. Cloud server architecture diagram


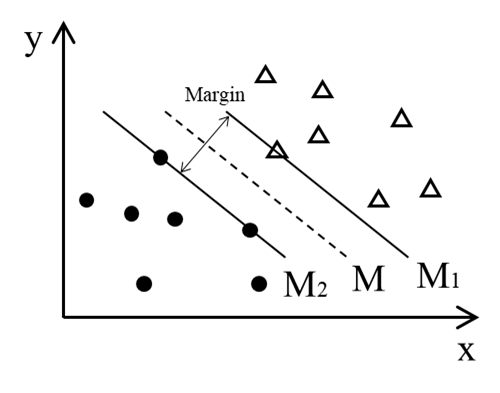


Figure 5. Schematic diagram of 2D linear separation


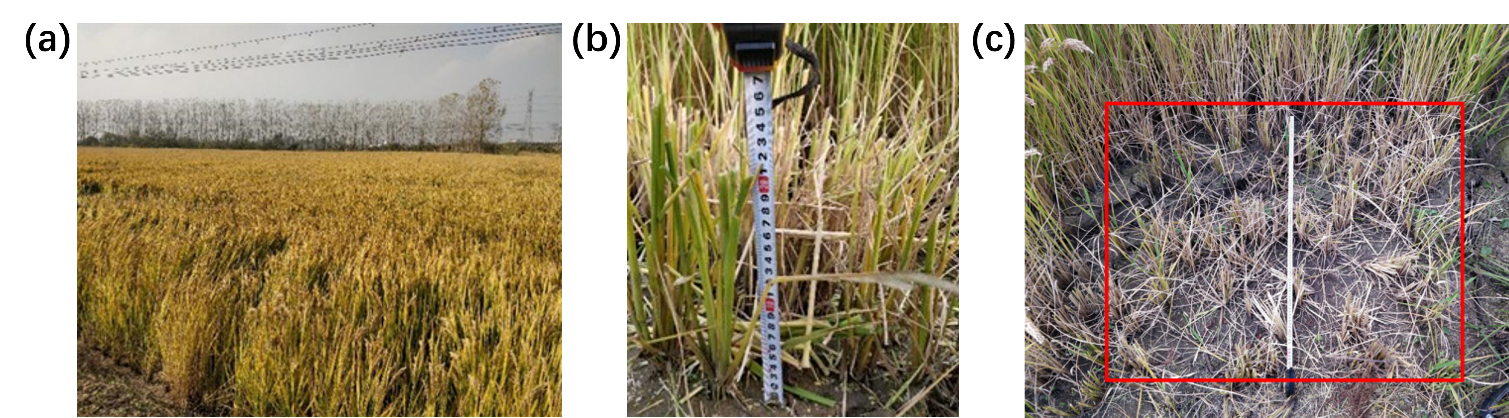


(a) General view of the experiment field; (b) Cutting height measurement; (c) Reaping area survey

Figure 6. Feed rate calibration experiment


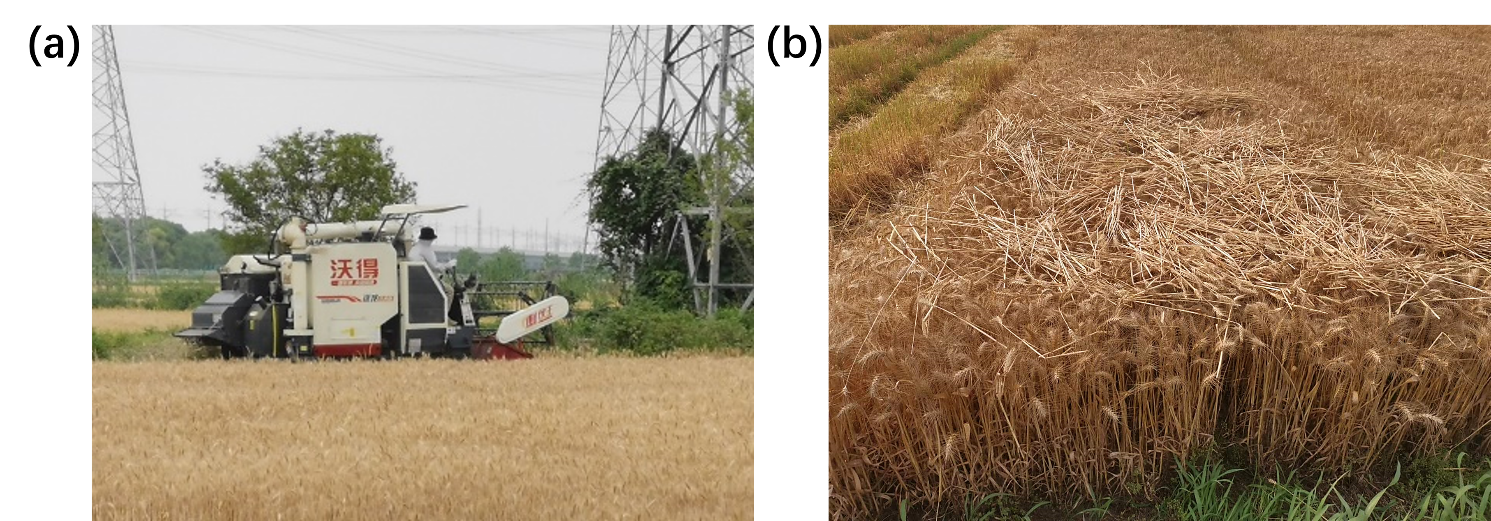


(a) Lower cutting table and increase speed; (b) Increase crop

Figure 7. Fault Setting experiment


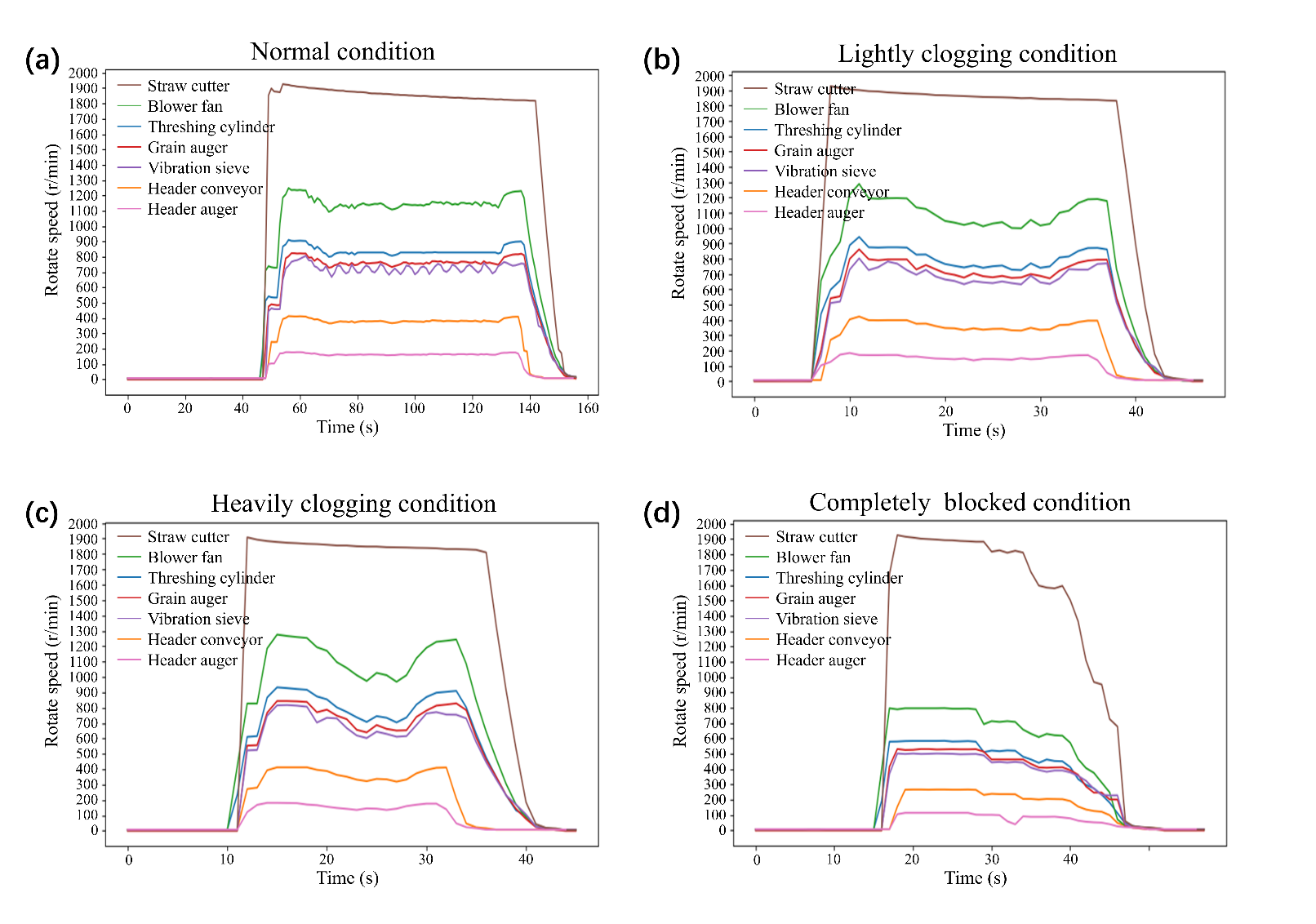


(a) Rotate speed in normal condition; (b) Rotate speed in lightly clogging condition; (c) Rotate speed in heavily clogging condition; (d) Rotate speed in completely blocked condition

Figure 8. Speed data of each part


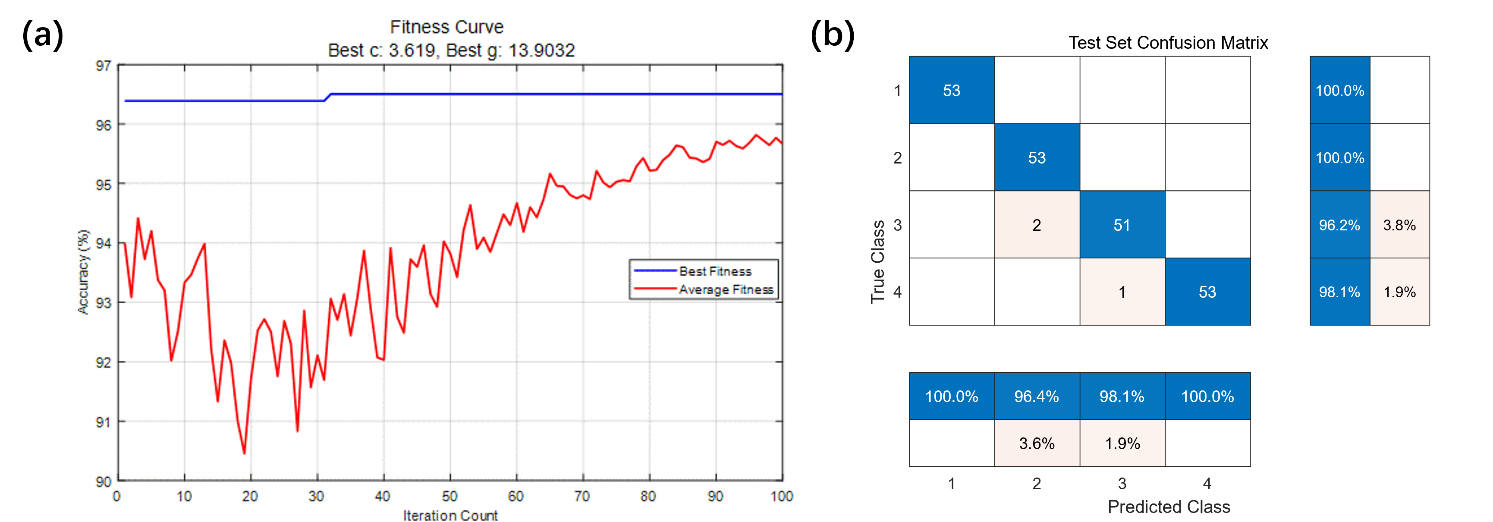


(a) Fitness change curve; (b) IPSO-SVM test set confusion matrix

Figure 9. Fault Setting experiment


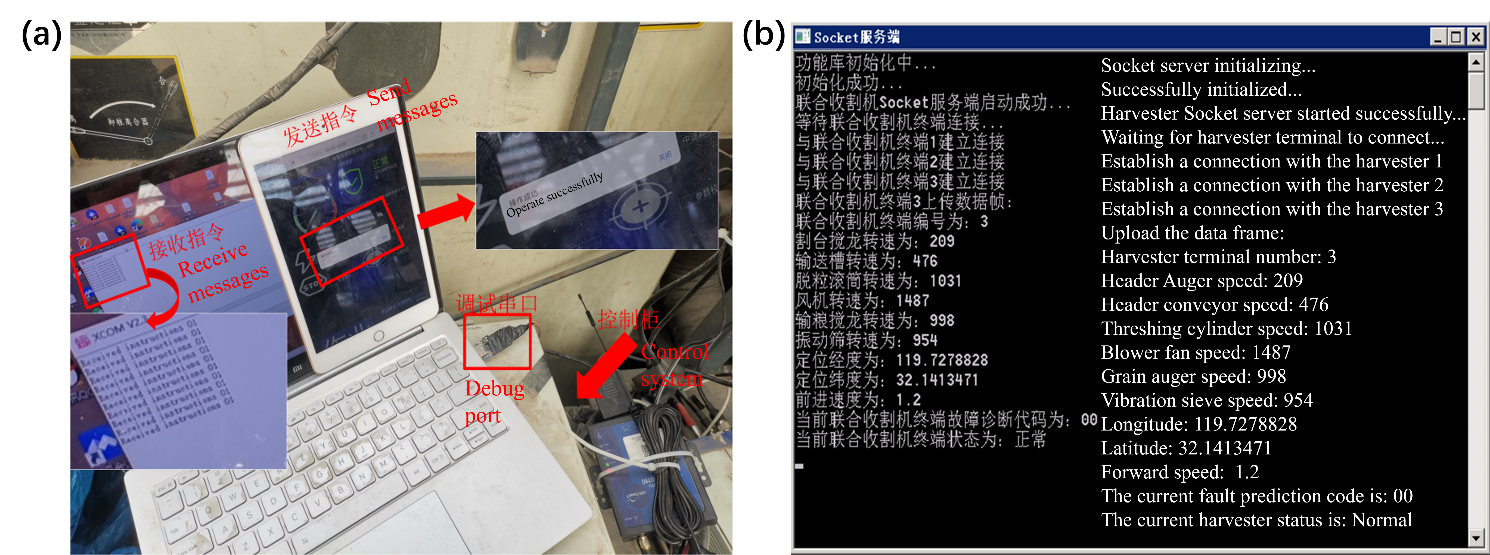


(a) Onboard terminal tests; (b) Server communication window

Figure 10. Cloud server communication experiment


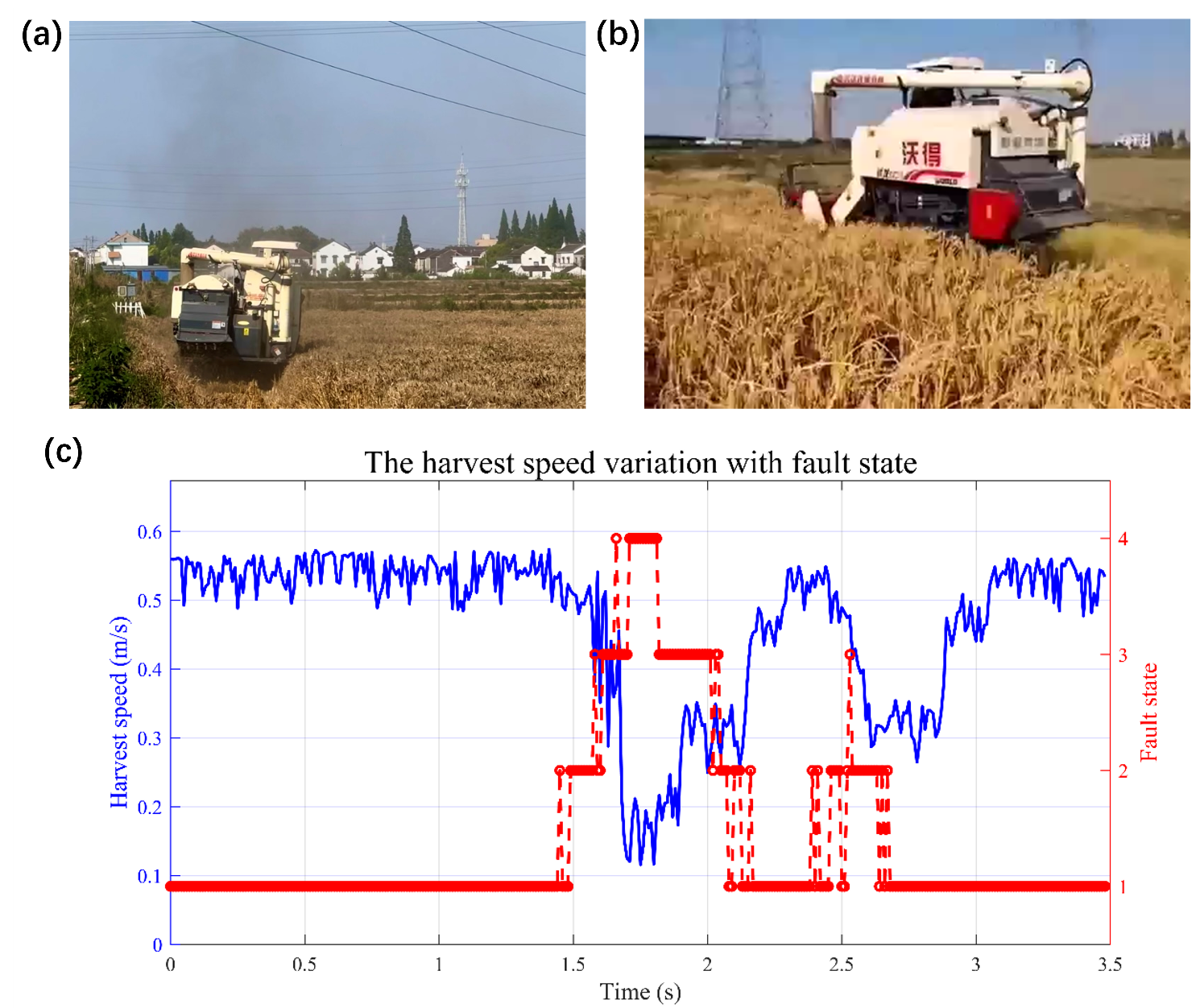


(a) Heavily clogging with black smoke caused by belt slippage; (b) Unmanned harvesting with speed regulation; (c) Plot of harvest speed variation with fault state

Figure 11. Fault prediction and speed regulation experiment

## Supplementary Tables

Table 1. The main parameters of the World Ruilong 4LZ-5.0

| Parameter | Value | Unit |
| --- | --- | --- |
| Combine dimensions (length- width- height) | 4960 - 3950 - 2830 | mm |
| Combine weight | 3000 | kg |
| Engine power | 75 | kw |
| Gear shift method | 3 speeds HST | - |
| Travel speeds (at rated engine speeds) | Low speed: 0 to 1 | m/sec |
|  | Standard: 0 to 1.5 |  |
|  | High speed: 0 to 2 |  |
| Cut width | 2200 | mm |
| Feeding capacity | 5 | kg/s |
| Operational efficiency | 0.68 – 0.85 | ha/h |
| Grain tank capacity | 1.5 | m^3^ |

Table 2. Fuzzy control rule

| Fault status | Feeding Intake | Speed |
| --- | --- | --- |
| N | S | F |
| N | M | M |
| N | L | S |
| LC | S | M |
| LC | M | S |
| LC | L | ST |
| HC | S | S |
| HC | M | ST |
| HC | L | ST |
| CB | Any | ST |

Table 3. Weighing results of crop blocks

| Crop number | Cutting height (m) | First weighing (kg) | Second weighing (kg) | Third weighing (kg) | Mass-area ratio (kg/m²) | Average mass-area ratio (kg/m²) |
| --- | --- | --- | --- | --- | --- | --- |
| 1 | 0.15 | 21.01 | 21.00 | 21.02 | 5.56 | 5.56 |
| 2 |  | 22.16 | 22.12 | 22.21 | 5.71 |  |
| 3 |  | 21.22 | 21.20 | 21.22 | 5.60 |  |
| 4 |  | 21.40 | 21.41 | 21.40 | 5.61 |  |
| 5 |  | 21.07 | 21.10 | 21.09 | 5.57 |  |
| 6 |  | 19.84 | 19.89 | 19.88 | 5.49 |  |
| 7 |  | 21.43 | 21.42 | 21.41 | 5.62 |  |
| 8 | 0.25 | 19.84 | 19.82 | 19.84 | 4.96 | 5.05 |
| 9 |  | 21.02 | 20.99 | 21.06 | 5.26 |  |
| 10 |  | 20.28 | 20.25 | 20.27 | 5.07 |  |
| 11 |  | 20.56 | 20.57 | 20.56 | 5.14 |  |
| 12 |  | 20.13 | 20.16 | 20.15 | 5.04 |  |
| 13 |  | 18.94 | 18.97 | 18.97 | 4.74 |  |
| 14 |  | 20.68 | 20.68 | 20.66 | 5.17 |  |
| 15 | 0.35 | 16.91 | 16.88 | 16.89 | 4.04 | 4.04 |
| 16 |  | 18.07 | 18.02 | 18.11 | 4.06 |  |
| 17 |  | 17.28 | 17.25 | 17.27 | 4.05 |  |
| 18 |  | 17.66 | 17.68 | 17.67 | 4.06 |  |
| 19 |  | 17.02 | 17.04 | 17.03 | 4.04 |  |
| 20 |  | 15.94 | 15.97 | 15.96 | 4.00 |  |
| 21 |  | 17.22 | 17.21 | 17.20 | 4.05 |  |

Table 4. Harvester status categories and classification labels

| Status category | Classification label |
| --- | --- |
| Normal  Lightly clogging | 1  2 |
| Heavily clogging | 3 |
| Completely blocked | 4 |

Table 5. Comparison Table of Classification Accuracy of Different Models

| Status category | SVM (%) | PSO-SVM (%) | IPSO-SVM (%) |
| --- | --- | --- | --- |
| Norma | 73.58 ± 5.30 | 86.79 ± 4.05  100.00 ± 1.12¹ | 100.00 ± 1.12¹  100.00 ± 1.12¹ |
| Lightly clogging | 100.00 ± 1.12¹ |  |  |
| Heavily clogging | 60.38 ± 5.86 | 88.68 ± 3.80 | 96.23 ± 2.27 |
| Completely blocked | 75.93 ± 5.10 | 85.19 ± 4.23 | 98.15 ± 1.61 |
| Total | 77.46 ± 2.51 | 90.15 ± 1.78 | 98.59 ± 0.71 |

1. For accuracies that are exactly 100.00%, the Rule of Three was applied, i.e., ±(3/n)×100%, to avoid a zero-width confidence interval under the normal approximation.
